# Development of an *in vitro* metabolic dysfunction-associated steatohepatitis model to investigate altered drug metabolizing enzymes, transport proteins, and hepatobiliary disposition

**DOI:** 10.3389/fphar.2025.1664808

**Published:** 2026-01-12

**Authors:** William A. Murphy, Sarina Kyburz, Henry Ho, Matthew Shane Loop, John K. Fallon, Jacqueline B. Tiley, Thomas Kralj, Kim L. R. Brouwer

**Affiliations:** 1 Division of Pharmacotherapy and Experimental Therapeutics, UNC Eshelman School of Pharmacy, University of North Carolina at Chapel Hill, Chapel Hill, NC, United States; 2 Biopharmacy, Department of Pharmaceutical Sciences, University of Basel, Basel, Switzerland; 3 Department of Health Outcomes Research and Policy, Harrison College of Pharmacy, Auburn University, Auburn, AL, United States; 4 Division of Pharmacoengineering and Molecular Pharmaceutics, and Center for Nanotechnology in Drug Delivery, UNC Eshelman School of Pharmacy, University of North Carolina at Chapel Hill, Chapel Hill, NC, United States; 5 Pharmaron Lab Services Inc., Germantown, MD, United States

**Keywords:** MASH, lysophospholipids, fatty liver disease model, lipid–cytokine treatments, intracellular lipid droplet formation, hepatobiliary transport, quantitative targeted absolute proteomics (QTAP), sandwich-cultured human hepatocytes (SCHH)

## Abstract

**Introduction:**

Metabolic dysfunction-associated steatotic liver disease (MASLD) is estimated to affect ∼30% of adults globally. The progressive form of MASLD, metabolic dysfunction-associated steatohepatitis (MASH), is a leading cause of chronic liver disease. MASH is marked by hepatocellular fat accumulation (steatosis), ballooning, and inflammation. Although many *in vitro* and *in vivo* models replicate MASH pathophysiology, no *in vitro* hepatocyte MASH model has been evaluated for its ability to reflect clinically observed changes in drug metabolizing enzymes (DMEs) and transporters. In this study, we addressed this gap by developing a model using sandwich-cultured human hepatocytes (SCHH) that mimics both MASH pathophysiology and alterations in DME and transporter concentrations and function.

**Methods:**

Lipid–cytokine treatments were first optimized using differentiated HuH-7 cells based on cellular toxicity and their ability to induce a MASH-like phenotype. Three final treatments—all including TNF-α (1 ng/mL) and IL-6 (1.2 ng/mL)—were selected for SCHH evaluation: (1) oleic acid (OA):palmitic acid (PA) (1:2, 0.5 mM), (2) a lipid mix (lysophospholipids mixture + OA:PA), and (3) lipid mix + 0.01 mM cholesterol. Treatments were incubated for 72 h with SCHH from three donors. Quantitative targeted absolute proteomics (QTAP) assessed the transporter and DME concentrations, whereas B-CLEAR^®^ technology evaluated transporter function using the probe substrates [^3^H]-taurocholate (TCA) and [^3^H]-estradiol-17β-glucuronide (E_2_17G).

**Results:**

All three treatments significantly increased lipid droplet formation and peroxidation in SCHH with minimal toxicity. These treatments also altered DME and transporter concentrations in a manner similar to the changes observed in liver tissue from patients with MASH. Across treatments, concentrations of the bile salt export pump (BSEP), sodium taurocholate co-transporting polypeptide (NTCP), organic anion transporting polypeptide (OATP) 1B1, OATP1B3, and multidrug resistance-associated protein (MRP) 2 were reduced by 0.66–0.57-fold, 0.71–0.52-fold, 0.74–0.63-fold, 0.82–0.80-fold, and 0.71–0.48-fold, respectively. Correspondingly, the TCA apparent uptake clearance and biliary clearance were reduced by 0.70–0.26-fold and 0.61–0.27-fold, respectively. E_2_17G apparent uptake clearance was reduced by 0.67–0.35-fold, whereas biliary excretion index values were reduced to negligible levels.

**Discussion:**

These findings demonstrate that lipid–cytokine treatments induce MASH-like changes in SCHH, including clinically relevant reductions in DME and transporter concentrations and function. This model may serve as a valuable tool for predicting altered hepatobiliary drug disposition in MASH.

## 1 Introduction

Metabolic dysfunction-associated steatotic liver disease (MASLD) is currently the most prevalent chronic liver disorder (Moon et al., 2020). MASLD development and progression to metabolic dysfunction-associated steatohepatitis (MASH) is influenced by a complex interaction of metabolic, genetic, and lifestyle-related factors (Powell et al., 2021). MASLD pathogenesis is initiated with excess lipid deposition in hepatocytes. Lipotoxicity can occur once lipid accumulation surpasses the liver’s metabolic capacity. This can trigger inflammation and subsequent cell death, tissue regeneration, and fibrogenesis (Powell et al., 2021). MASH is the progressive form of MASLD. Lobular inflammation, hepatocellular ballooning, and/or fibrosis observed on liver biopsy are hallmarks of MASH (Loomba et al., 2021).

MASLD can impact the clinical pharmacokinetics of drugs by altering hepatic drug-metabolizing enzymes (DMEs) and transport proteins (Murphy et al., 2023). This may have important implications for the efficacy and safety of medications prescribed to patients with MASLD (Patel et al., 2017; Desai et al., 2018; Marie et al., 2023). The increasing global prevalence of MASLD prompts an urgent need to gain more insight into the influence of MASLD on drug pharmacokinetics and therapeutic response in patients with this disease. Although there is currently a basic understanding of how MASLD may influence drug pharmacokinetics (Murphy et al., 2023), *in vitro* systems designed specifically to evaluate hepatobiliary drug transport and disposition in a MASLD-like physiologic environment are less well established. This is an important knowledge gap because clinical studies designed specifically to assess the impact of MASLD on drug disposition are costly, and such studies may be challenging to undertake (Bentley et al., 2019).

Sandwich-cultured human hepatocytes (SCHH) are a well-established *in vitro* system to evaluate hepatobiliary drug transport and disposition (Swift et al., 2010). Modification of SCHH to mimic a MASH-like phenotype has been explored previously using fatty acid (FA)-enriched culture media (Kralj et al., 2022). This model successfully replicated elevated glycerolipid levels, as observed in hepatocytes from patients with MASH (Kralj et al., 2022). However, total phospholipid levels and individual phospholipids [e.g., phosphatidylcholine (PC), phosphatidylethanolamine (PE), phosphatidylglycerol, phosphatidylinositol, and phosphatidylserine] were not increased. Furthermore, hepatobiliary drug transport proteins and DMEs were not evaluated in this model.

Many preclinical and *in vitro* models have been developed to explore the pathophysiology of MASLD/MASH and develop new drug treatments targeting MASLD initiation and progression based on the current understanding of this disease (Soret et al., 2021; Ramos et al., 2022; Gatzios et al., 2022; Ströbel et al., 2021; Gallage et al., 2022; Santhekadur et al., 2018; van Riet et al., 2024; Kwon et al., 2025; Odanga et al., 2024). Although previous *in vitro* MASLD models have been used to assess DMEs and drug-induced toxicity (Kozyra et al., 2018; Begriche et al., 2023), thorough characterization of drug transporters and evaluation of hepatobiliary drug disposition are lacking. The primary objective of this study was to develop an *in vitro* MASH model to study MASH-mediated changes in hepatobiliary drug disposition using SCHH that recapitulate pathophysiologic/phenotypic (e.g., hepatocyte lipid droplet formation), transporter, and DME alterations observed in the livers of patients with MASH. SCHH were selected as the cell model for this study because they are well characterized in terms of transporter and DME concentrations and function, and established assays are available to evaluate hepatic drug disposition (De Bruyn et al., 2013; Yang et al., 2016). To achieve this objective, an *in vitro* MASH-like phenotype with minimal cell death or toxicity was induced in SCHH using media supplementation/treatment. The following treatment components were selected based on their reported roles in MASLD progression or observed increases in the systemic circulation and/or liver tissue of MASH patients: free FAs (Kartsoli et al., 2020; Kralj et al., 2022), lysophospholipids (Kartsoli et al., 2020; Yamamoto et al., 2022; Kakisaka et al., 2012), free cholesterol (Horn et al., 2022; Puri et al., 2007), and pro-inflammatory cytokines (Duan et al., 2022). Lysophospholipids were chosen because they are the primary form of phospholipids that are absorbed in the intestine and introduced to the liver *via* the portal circulation (Hui, 2016). Considering the “multiple-hit” hypothesis for progression from metabolic dysfunction-associated steatotic liver (MASL) to MASH (Buzzetti et al., 2016; Peng et al., 2020), treatments were designed to induce intracellular lipid accumulation (i.e., free FAs, lysophospholipids, and free cholesterol). In addition, tumor necrosis factor (TNF)-α and interleukin (IL)-6 were included to activate the hepatocyte inflammasome (Szabo and Petrasek, 2015). TNF-α and IL-6 were selected as the pro-inflammatory cytokines for this treatment due to their proposed roles in MASH development and progression (Fontes-Cal et al., 2021; Duan et al., 2022; Lu et al., 2022; Vachliotis and Polyzos, 2023). The inclusion of these cytokines was a viable option to induce cellular inflammation because human hepatocytes express receptors for TNF-α and IL-6 (Shuh et al., 2013; Schmidt-Arras and Rose-John, 2016). The direct addition of cytokines was necessary because the cell culture systems utilized in the present study did not contain non-parenchymal or immune-related cells.

The free FAs, namely, oleic acid (OA) and palmitic acid (PA), were used in a 1:2 ratio (167 μM OA:333 μM PA), as described by Kralj et al. (2022). Lysophospholipids were also utilized. A 2:1 ratio of lysophosphatidylcholines (LysoPCs) to lysophosphatidylethanolamines (LysoPEs) was selected based on previous findings of increased LysoPCs (Puri et al., 2007) and decreased LysoPEs (Gorden et al., 2015) in MASH liver samples. Additionally, a 2:1 concentration ratio of unsaturated to saturated lysophospholipids with a total lysophospholipid concentration of 11.25 μM was used based on solubility limitations. Free cholesterol levels in livers from patients with MASH are reported to be 12.9 mM (Puri et al., 2007), whereas normal free cholesterol levels in the blood are estimated to be 4.3 mM (Estronca et al., 2014). However, initial testing showed that free cholesterol concentrations of 0.25 mM or higher caused cellular toxicity when combined with TNF-α and IL-6 in the selected cell systems (see Section 3.1). Therefore, all subsequent experiments were conducted with free cholesterol concentrations below 0.25 mM. The initial concentrations tested for TNF-α and IL-6 were selected based on a prior study employing primary human hepatocytes (Gramignoli et al., 2022) and optimized based on the toxicity data in the present study. A 1.2:1 concentration ratio of IL-6 to TNF-α was informed by clinical data in MASH (Ajmera et al., 2017).

In this study, we established an *in vitro* MASH model using cryopreserved primary human hepatocytes cultured in sandwich configuration. Various cell culture treatments were first optimized using a cost-effective and high-throughput differentiated human hepatoma (HuH)-7 cell system (Saran et al., 2022a) before evaluation in SCHH. Then, SCHH were characterized to identify treatment conditions that best recapitulate the molecular and phenotypic features of MASH. Lipid–cytokine treatments showing the closest resemblance to the disease phenotype were subsequently examined for their effects on drug transporter and DME concentrations using quantitative targeted absolute proteomics (QTAP). Finally, the impact of these treatments on the hepatobiliary disposition of select transporter probe substrates (i.e., taurocholate and estradiol-17β-glucuronide) was evaluated for the first time using B-CLEAR^®^ technology (Swift et al., 2010; Liu et al., 1999). These studies provide the foundation for using this *in vitro* MASH model in SCHH to predict alterations in hepatobiliary drug disposition in patients with MASH.

## 2 Materials and methods

### 2.1 Chemicals and materials

Water-soluble oleic acid (OA; catalog #O1257-10 mg), water-soluble free cholesterol (#C4951-30 MG), staurosporine (#S6942), fetal bovine serum (FBS; #F2442), taurocholic acid (TCA; #T4009), and estradiol-17β-glucuronide (E_2_17G; #E1127) were purchased from Sigma-Aldrich (St. Louis, MO). Sterile water was used to dissolve the water-soluble compounds. 1-Oleoyl-2-hydroxy-sn-glycero-3-phosphoethanolamine (18:1 LysoPE; #846725), 1-oleoyl-2-hydroxy-sn-glycero-3-phosphocholine (18:1 LysoPC; #845875), 1-palmitoyl-2-hydroxy-sn-glycero-3-phosphoethanolamine (16:0 LysoPE; #856705), and 1-palmitoyl-2-hydroxy-sn-glycero-3-phosphocholine (16:0 LysoPC; #855675) were purchased from Avanti Polar Lipids (Alabaster, AL). Tumor necrosis factor alpha (TNF-α; #300-01A) and interleukin-6 (IL-6; #200–06) were purchased from PeproTech (Cranbury, NJ). Palmitic acid (PA; #10006627), dexamethasone (#11015), and imatinib (#13139) were purchased from Cayman Chemicals (Ann Arbor, MI). BODIPY™ 493/503 (#D3922), BODIPY™ 558/568 C_12_ (#D3835), BODIPY™ 665/676 (#B3932), MitoTracker™ Orange CM-H2TMRos (#M7511), CellEvent™ Caspase-3/7 Detection Reagent (#C10423), Hoechst Blue 33342 Solution (#62249), Dulbecco’s modified Eagle’s medium (DMEM: #11995-065), Williams’ Medium E without Phenol Red (#A1217601), penicillin–streptomycin (#15140122), Ca^2+^- and Mg^2+^-free Dulbecco’s phosphate buffered saline (PBS; #14190144), Hank’s Balanced Salt Solution (standard HBSS; #14025–092), and Ca^2+^- and Mg^2+^-free HBSS (Ca^2+^-free HBSS; #14175–095) were purchased from Thermo Fisher Scientific (Waltham, MA). Anhydrous ≥99.9% dimethyl sulfoxide (DMSO; #D12345) used in the dissolution of hygroscopic compounds (e.g., lysophospholipids, BODIPY™ probes) was purchased from Thermo Fisher Scientific, and ≥99.5% DMSO (#41639) used for cell culture was purchased from Sigma-Aldrich. Cryopreserved Transporter Certified™ human hepatocytes from three different donors (JEL, WID, and IWM; Supplementary Table 1) and QualGro™ seeding, thawing, overlay, and maintenance media were purchased from BioIVT (Baltimore, MD). Collagen I-coated BioCoat™ plates (24-well; #356408) were purchased from Corning Life Sciences (Tewksbury, MA). [^3^H]-TCA (#NET322250UC, 6.5 Ci/mmol, radiochemical purity >97%), and [^3^H]-E_2_17G (#NET1106250UC, 52.9 Ci/mmol, radiochemical purity >97%) were purchased from PerkinElmer Life Sciences (Boston, MA).

### 2.2 Cell culture treatment design for the induction of a MASH-like phenotype

Table 1 summarizes all the selected treatment components and the corresponding concentrations evaluated within each *in vitro* system. Lysophospholipid notation “18:1” indicates a fatty acid chain of 18 carbon atoms with one double bond, whereas “16:0” indicates a fatty acid chain of 16 carbon atoms with zero double bonds. A final media concentration of 0.5% DMSO was selected to limit any potential impact on human hepatocyte gene expression or viability (Sumida et al., 2011). OA, PA, and lysophospholipid concentrations were kept constant and are collectively referred to as the “lipid mix.” All treatments were included in the cell culture media for 72 h before endpoint assays, which is a previously optimized duration between collagen overlay and functional assay assessment in SCHH (Yang et al., 2016). Positive toxicity controls (staurosporine [500 nM] for differentiated HuH-7 cell culture and imatinib [40 μM] for SCHH) were incubated in the cell culture media for 24 h before endpoint assays.

**TABLE 1:** Concentration ranges of individual treatment components tested in the corresponding culture system.

Treatment component	Concentration(s) tested	*In vitro* cell culture model
Oleic acid^a^	167 μM	Differentiated HuH-7 cells
		SCHH
Palmitic acid^a^	333 μM	Differentiated HuH-7 cells
		SCHH
1-Oleoyl-2-hydroxy-sn-glycero-3-phosphoethanolamine (18:1 LysoPE)^a^	2.5 μM	Differentiated HuH-7 cells
		SCHH
1-Oleoyl-2-hydroxy-sn-glycero-3-phosphocholine (18:1 LysoPC)^a^	5 μM	Differentiated HuH-7 cells
		SCHH
1-Palmitoyl-2-hydroxy-sn-glycero-3-phosphoethanolamine (16:0 LysoPE)^a^	1.25 μM	Differentiated HuH-7 cells
		SCHH
1-Palmitoyl-2-hydroxy-sn-glycero-3-phosphocholine (16:0 LysoPC)^a^	2.5 μM	Differentiated HuH-7 cells
		SCHH
Free cholesterol	0.01 mM–0.5 mM	Differentiated HuH-7 cells
	0.01 mM–0.25 mM	SCHH
IL-6	1.2 ng/mL – 30 ng/mL	Differentiated HuH-7 cells
	1.2 ng/mL – 15 ng/mL	SCHH
TNF-α	1 ng–25 ng/mL	Differentiated HuH-7 cells
	1 ng/mL – 12.5 ng/mL	SCHH

Lysophospholipid notation “18:1” indicates a fatty acid chain of 18 carbon atoms with one double bond; “16:0” indicates a fatty acid chain of 16 carbon atoms with zero double bonds.

^a^ Contained in the lipid mix.

HuH-7, human hepatoma; IL, interleukin; LysoPC, lysophosphatidylcholine; LysoPE, lysophosphatidylethanolamine; SCHH, sandwich-cultured human hepatocytes; TNF, tumor necrosis factor.

### 2.3 Optimization of the *in vitro* MASH culture model using differentiated HuH-7 cells

The initial optimization of the *in vitro* MASH culture model utilized a previously described differentiated HuH-7 cell system that displays hepatocyte-like morphology and bile canalicular-like formation, and is amenable to economical high-throughput testing (Saran et al., 2022a). The HuH-7 cell line (JCRB0403) was acquired from Sekisui Xenotech. Cell line identity was confirmed by the UNC Vironomics Core. HuH-7 cell cultures were maintained and passaged in T-25 (Sarstedt [Newton, NC] #83.3910) or T-75 (Sarstedt #83.3911) cell culture flasks using maintenance media (DMEM supplemented with 10% FBS and 1% penicillin–streptomycin) until fully confluent. On culture day 0, HuH-7 cells were seeded at 0.075 million cells/well in a 96-well collagen type-I-treated opaque-walled plate (Greiner Bio-One #655956). On culture day 2, maintenance medium was supplemented with 1 μM dexamethasone and 0.5% DMSO (differentiation media). On culture day 7, the cells were overlaid with 0.25 mg/mL Matrigel Basement Membrane Matrix Phenol Red-Free (Corning #356237, lot #3068002) in ice-cold differentiation media. The culture was then maintained for one additional week, with differentiation media replaced every 2–3 days. To ensure optimal hepatocyte-like characteristics, MASH culture treatments were initiated on day 14 of culture. This occurred 1 week after Matrigel overlay (Saran et al., 2022a). Additional amounts of DMSO were added to each treatment to ensure a final working concentration of 0.5% for comparison to the non-MASH-inducing treatment control group that contained 0.5% DMSO in differentiation media. Treatment and control differentiation media were replaced every 24 h for 72 h on culture days 14–16. All terminal cellular toxicity and phenotypic endpoints were assessed on day 17 of culture. All HuH-7 cell cultures were between passage numbers 16–24.

### 2.4 Optimization of the *in vitro* MASH culture model using sandwich-cultured human hepatocytes (SCHH)

Following the identification of optimal treatment concentrations using the differentiated HuH-7 cell culture model, MASH culture treatments were tested in SCHH. On culture day 0, cryopreserved Transporter Certified^®^ human hepatocytes were thawed using QualGro™ thawing media, diluted to 0.9–1.0 million cells/mL in QualGro seeding media, and seeded at a density of 0.45–0.5 million cells/well in 24-well BioCoat Collagen-I-coated plates. Approximately 16 h–20 h following seeding (culture day 1), cells were overlaid with 0.25 mg/mL of Matrigel Basement Membrane Matrix Phenol Red-Free (Corning #356237, lots #3068002, 3068003) in ice-cold QualGro maintenance media. The following day (culture day 2), treatments were dissolved in QualGro maintenance media supplemented with additional DMSO to reach a final working concentration of 0.5%. Treatments and control QualGro maintenance media supplemented with 0.5% DMSO were administered to SCHH on the same day. Treatment and control maintenance media were exchanged every 24 h on culture days 2–4. All terminal cell toxicity and phenotypic endpoints were assessed on culture day 5.

### 2.5 Phenotypic endpoint assessment

Following the culmination of the 72-h treatment on culture day 17 (differentiated HuH-7 cells) or day 5 (SCHH), phenotypic endpoints were assessed using fluorescent probes. Given their documented roles in the progression of MASL to MASH (Ramanathan et al., 2022; Pierantonelli and Svegliati-Baroni, 2019), the following four endpoints (fluorescent probes) were evaluated:

1. Intracellular neutral lipid accumulation/lipid droplet formation (BODIPY™ 493/503).
2. Cellular uptake and trafficking of free FAs (BODIPY™ 558/568 C_12_).
3. Intracellular free radical lipid peroxidation (BODIPY™ 665/676).
4. Mitochondrial function/stress (MitoTracker).

A Hoechst Blue 33342 stain was used to identify cell nuclei. Fluorescent probes were evaluated using fluorescence-based high-content imaging (HCI) with the CellInsight™ CX7 LZR High Content Analysis platform (Thermo Fisher Scientific #CX7A1110LZR). HCI was performed using a 10X imaging objective, with the HCS Studio™ Cell Analysis Software (Thermo Fisher Scientific; version 6.6.1) used for cell identification, subsequent fluorescence object detection/surface rendering, and automated data analysis. Specifically, the fluorescence signal from each probe was rendered into a two-dimensional object. The Hoechst Blue nuclei stain was used to detect cell nuclei, followed by the application of an optimized software-based algorithm to estimate the surrounding cell area. All fluorescence-based HCI data are reported as the mean rendered surface area (μm^2^) per cell or the mean rendered object count per cell (see caspase-3/7 detection assay below).

To perform fluorescent probe and Hoechst Blue staining, treatment and control media were aspirated on the terminal culture day and cells were washed twice with PBS. Fluorescent probe solutions (Supplementary Table 2) were freshly prepared in FBS-free DMEM (for differentiated HuH-7 cells) or Williams’ Medium E (for SCHH) and added to the cells at 37 °C/5% CO_2_ for 30 min–45 min with the plate covered in tin foil to minimize light exposure. For the BODIPY™ 558/568 C_12_ probe, cells were incubated in the absence of culture treatments for 24 h to allow for adequate FA cellular uptake per manufacturer instructions. Then, the FBS-free dosing solutions were aspirated and replaced with FBS-free media and immediately imaged using appropriate laser emission and excitation wavelengths (Supplementary Table 2). In differentiated HuH-7 cells, six replicate measures were obtained for each treatment and control group, whereas in SCHH, three replicate measures per donor were obtained.

### 2.6 Cellular toxicity assessment

Early-stage cellular apoptosis was assessed using a CellEvent™ Caspase-3/7 Detection Reagent with the CellInsight™ CX7 High-Content Screening platform (10X objective) using the same fluorescent probe staining and imaging protocol described above. The PBS wash step was omitted to prevent the detachment of apoptotic cells, and the probe solution was incubated for 45 min as per manufacturer instructions. Cell viability/ATP content was determined with the CellTiter-Glo^®^ Luminescent Cell Viability Assay (Promega #G7570) using a PowerWave XS Microplate spectrophotometer (BioTek Instruments). A predetermined “threshold” luminescence value relative to the control of 70% was used to define the undesirable cellular toxicity for this assay. Although no toxicity threshold values have been established specifically for the ATP assay, a cell viability threshold of at least <75% was used to avoid false positives when assessing cytotoxicity based on previous literature (Azqueta et al., 2022). In differentiated HuH-7 cells, six replicate measures were obtained for each treatment and control group, whereas in SCHH, three replicate measures per donor were obtained.

For toxicity endpoints, a one-way ANOVA adjusted for multiple comparisons using Dunnett’s test was performed to compare each treatment group to the control. For phenotypic endpoints evaluated in HuH-7 cells, a one-way ANOVA with a *post hoc* Tukey’s test was used to facilitate comparison across all treatment groups. Specifically, after confirming an ANOVA F statistic of *p* < 0.05, comparisons between treatments with and without cytokines and different concentrations of cholesterol in the presence of the lipid mix were evaluated for significance (*p* < 0.05). Statistical testing was performed in GraphPad Prism (version 10.1.2). For phenotypic endpoints in SCHH, a linear mixed-effects model was fit for each outcome with a random slope on hepatocyte donor to account for inter-donor variability. Models describing the lipid droplet formation outcome (BODIPY™ 493/503) were fit using a gamma distribution and log link function (*lme4* package (Bates et al., 2015)) in RStudio (R version 4.3). Models describing the lipid peroxidation outcome (BODIPY™ 558/568) were fit using a Gaussian distribution. When comparing treatments with and without cytokines for both outcomes, models were fit using a Gaussian distribution.

### 2.7 Quantitative targeted absolute proteomics (QTAP)

SCHH membrane protein fractions were extracted following the 72-h treatment period (culture day 5) for QTAP analysis using a previously described differential detergent fractionation (DDF) method (Qasem et al., 2021). Briefly, SCHH cultured at 450,000 cells/well were washed twice with 1 mL of PBS containing 1 mM phenylmethylsulfonyl fluoride. To release non-membrane bound intracellular content, 250 μL of digitonin buffer (Qasem et al., 2021) was added to each well, and the cells were incubated at 4 °C for 10 min under gentle agitation. The digitonin buffer, now enriched with cytosolic proteins (cytosolic protein fraction), was transferred to 1.5 mL tubes and stored at −80 °C (no analysis was performed on the cytosolic protein fraction in this study). To extract the membrane fraction, 150 μL of Triton X-100 buffer (Qasem et al., 2021) was added to each well and incubated at 4 °C for 30 min under gentle agitation. The Triton X-100 buffer, now enriched with cellular membrane proteins, was collected. The Pierce™ bicinchoninic acid Protein Assay Kit (#23225, Thermo Fisher Scientific) was used to quantify the total amount of protein in each sample. Membrane protein recovery per 450,000 cells ranged as follows for each hepatocyte donor across all sample treatments and replicates: 57.9 μg–68.0 μg (JEL), 70.7 μg–77.3 μg (WID), and 39.0 μg–56.6 μg (IWM). Ultimately, 20 μg of membrane protein was used from each sample for QTAP analysis. Triplicate samples were obtained for each treatment (three lipid–cytokine treatments + non-treated control [0.5% DMSO]) and donor (JEL, WID, and IWM) combination (12 samples per donor).

As previously described (Khatri et al., 2019; Fashe et al., 2022; Fallon et al., 2013), membrane protein was mixed with stable isotope labeled (SIL) proteotypic human peptide standards representing the transport and DME proteins of interest. The mixture was digested with trypsin (1:20 trypsin: protein ratio) and then recovered using solid-phase extraction. For QTAP analysis, 0.08 μg protein (0.2 µL volume) of each 20 µg digest was injected onto the micro LC-MS/MS system. The system consisted of an M-Class Acquity (Waters, Milford, MA) (microflow LC) coupled to a SCIEX (Framingham, MA) Triple Quadrupole 7500 mass spectrometer operated in the positive multiple reaction monitoring (MRM) mode. The M-Class Acquity trapping column was a Waters nanoEase™ M/Z Symmetry C18, 100Å, 5 μm, 180 μm × 20 mm column (part no. 186008821), and the analytical column was a Waters nanoEase™ M/Z Peptide BEH C18, 130Å, 1.7 µm, 300 μm × 100 mm column (part no. 186009258). The injection run time was 35 min. A QTAP laboratory human liver microsomes quality control (Gentest™; pool of 50) (Discovery Life Sciences, Huntsville, AL) was analyzed with the samples to ensure batch validity. MRM peak areas were determined by Analytics software embedded in SCIEX OS software (version 3.1.6.44), which was used for system control of the instrumentation. The SIL peptides used to report the protein concentration in the analyses and the MRMs for each (two MRMs for unlabeled [endogenous] peptides and two for each corresponding SIL peptides) are shown in Supplementary Table 3. These peptides have been used in previous studies (Khatri et al., 2019; Ohtsuki et al., 2012; Fallon et al., 2013; Fashe et al., 2022). Equal response of the unlabeled and SIL peptides was assumed. The ratio of the sum of the unlabeled MRM peak areas to the sum of the SIL MRM peak areas was calculated, and this was used to determine the peptide concentration based on the amount of SIL peptide added (0.5 pmol of each). For the bile salt export pump (BSEP) peptide, only one MRM transition was adequately detected for some samples from donors JEL and WID. For these samples, the peak area ratio based on that one MRM (unlabeled and SIL) was used to calculate the peptide concentration. Data for other representative peptides (most often one extra peptide) for many of the proteins were also acquired (*data not shown*), and these data were used as confirmatory data. Proteomics data are available in ProteomeXchange via the proteomics identification (PRIDE) database (Perez-Riverol et al., 2022) with accession number PXD071083. The lower limit of quantification (LLOQ) was set at 0.1 pmol/mg protein. For data analysis, protein concentrations between 0.1 pmol/mg protein and 0.02 pmol/mg protein (the lower limit of detection) were reported as determined, whereas concentrations below 0.02 pmol/mg protein were imputed as 0.02 pmol/mg protein. RStudio (version 2024.04.2 + 764; R version 4.4) was used to convert the peak area ratios to peptide (and, therefore, protein) concentrations (pmol/mg protein). To assess treatment-specific effects on transporter and DME protein concentrations, a linear mixed-effects model was fit for each protein with a random intercept on hepatocyte donor to account for inter-donor variability. Where appropriate, models were fit using a Gaussian distribution (*lme4* package (Bates et al., 2015)) in RStudio (R version 4.3), whereas *p* values, model predicted mean values, and 95% confidence intervals for each treatment and control group were extracted using the *lmerTest* package (Kuznetsova et al., 2017). For some of the analytes, the donor effects were so strong that a linear mixed-effects model created negative concentration predictions. For those models, we shifted to using a log link function for the linear mixed models, as opposed to the typical identity link function. Gaussian or gamma distributions were used for the models depending on the distribution of the raw data. GraphPad Prism was used to plot the raw data along with the model-derived predicted mean values and 95% confidence intervals; *p* < 0.05 was used to define statistical significance. Using model-predicted mean values, the mean fold-change across three hepatocyte donors compared to the control (0.5% DMSO) was determined and converted to log_2_ fold-change for additional comparisons.

### 2.8 Assessment of transporter function

Following the 72-h treatment period on culture day 5, the biliary excretion index (BEI) of [^3^H]-TCA and [^3^H]-E_2_17G in SCHH was measured using B-CLEAR^®^ technology (Swift et al., 2010; Liu et al., 1999; Ito et al., 2020). Standard HBSS, termed “cells + bile” or plus (+) buffer, contained Ca^2+^ and Mg^2+^, supporting normal cellular functions, whereas Ca^2+^-free HBSS, termed “cells” or minus (−) buffer, disrupts tight junctions. As described previously (Saran et al., 2022b), cells underwent two washes with either standard or Ca^2+^-free HBSS, with the latter containing 1 mM egtazic acid, before pre-incubation for 10 min at 37 °C. Post-incubation, cells were treated with 2 μM of [^3^H]-TCA (200 nCi/mL) or 0.3 μM of [^3^H]-E_2_17G (2 μCi/mL) for 10 min at 37 °C in standard HBSS, followed by three washes in ice-cold HBSS before freezing at −20 °C. Cell lysis was then performed with 0.5% Triton X-100% and 0.005% Antifoam-A in PBS. Radioactivity of cell lysates was measured using a Bio-Safe II counting cocktail (Research Products International; Mount Prospect, IL) and the Tri-Carb 3100 TR liquid scintillation analyzer (PerkinElmer Life Sciences). The total protein content was determined using the Pierce bicinchoninic acid Protein Assay Kit. [^3^H]-TCA and [^3^H]-E_2_17G accumulation in SCHH was normalized to the protein content. The BEI (Equation 1), apparent *in vitro* uptake clearance (CL_uptake,app_) (Equation 2), and apparent *in vitro* biliary clearance (CL_biliary,app_) (Equation 3) were calculated as follows:

$$BEI\;\left(\%\right)=\frac{{Accumulation}_{\left(Cells+Bile\right)}-{Accumulation}_{\left(Cells\right)}}{{Accumulation}_{\left(Cells+Bile\right)}}\;X\;100$$ (1)

$${CL}_{uptake,app}=\frac{{Accumulation}_{\left(Cells+Bile\right)}}{IncubationTime\;x\;{Concentration}_{media}}$$ (2)

$${CL}_{biliary,app}=\frac{{Accumulation}_{\left(Cells+Bile\right)}-{Accumulation}_{\left(Cells\right)}}{IncubationTime\;x\;{Concentration}_{media}}$$ (3)

Triplicate samples were obtained for each treatment (three lipid–cytokine treatments + non-treated control [0.5% DMSO]) and donor (JEL, WID, and IWM) combination (12 samples per donor). A one-way ANOVA adjusted for multiple comparisons using Tukey’s HSD test was performed across treatments for each donor.

## 3 Results

### 3.1 MASH-inducing treatments in differentiated HuH-7 cell culture model

**FIGURE 1:**
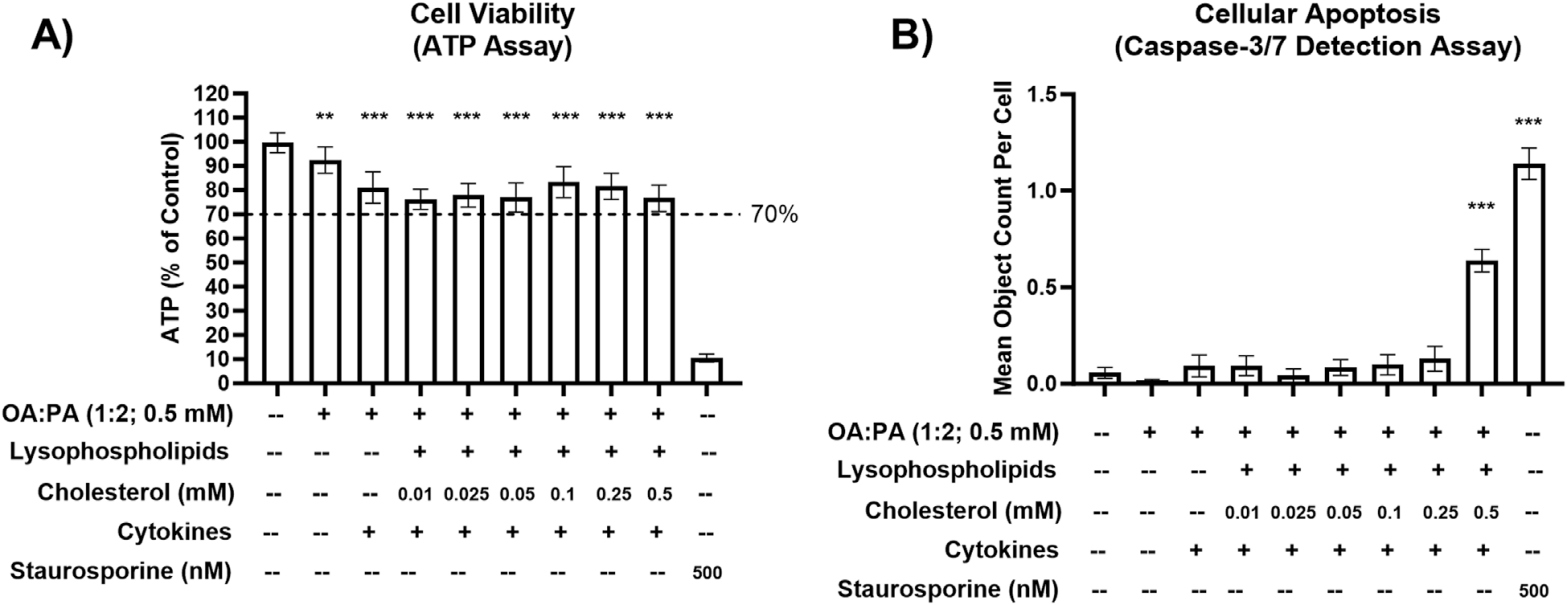
Cellular toxicity of various lipid–cytokine treatments in a differentiated HuH-7 cell culture model. **(A)** Cell viability (ATP) and **(B)** cellular apoptosis (caspase-3/7) following 72-h incubation of various lipid treatments with and without cytokines, and untreated control (0.5% DMSO), in differentiated HuH-7 cell culture. A pre-established “threshold” ATP luminescence value relative to the control of 70% was used to define undesirable cellular toxicity. Differentiated HuH-7 cells were treated with staurosporine (500 nM) for 24 h as a positive toxicity control. A one-way analysis of variance was performed for both endpoints and corrected for multiple comparisons using Dunnett’s test; **p < 0.01; ***p < 0.001 vs. control. Six replicate measures were available for each treatment and control group (mean ± standard deviation). Lysophospholipids comprised 2.5 uM 18:1 lysophosphatidylethanolamine (LysoPE) + 5 μM 18:1 lysophosphatidylcholine (LysoPC) + 1.25 μM 16:0 LysoPE +2.5 μM 16:0 LysoPC. Lysophospholipid notation “18:1” indicates a fatty acid chain of 18 carbon atoms with one double bond; “16:0” indicates a fatty acid chain of 16 carbon atoms with zero double bonds. Cytokines comprised 12.5 ng/mL TNF-α and 15 ng/mL IL-6. *DMSO, dimethyl sulfoxide; IL, interleukin; OA, oleic acid; PA, palmitic acid; TNF, tumor necrosis factor.*

Various concentrations of free cholesterol, TNF-α, and IL-6 were investigated independently and in combination with each other and the lipid mix in differentiated HuH-7 cells (Table 1). Despite TNF-α and IL-6 demonstrating minimal toxicity at respective concentrations of 25 ng/mL and 30 ng/mL in the HuH-7 cells (Supplementary Figure 1), the concentrations of 12.5 ng/mL and 15 ng/mL were used for initial phenotypic endpoint assessment due to a previous report of cytokine toxicity in primary human hepatocytes at concentrations of 20 ng/mL or higher (Gramignoli et al., 2022). Free cholesterol at a concentration of 0.5 mM in combination with cytokines and the lipid mix significantly increased cellular apoptosis in differentiated HuH-7 cells despite ATP levels remaining above the 70% threshold with this treatment (Figure 1). Therefore, cholesterol concentrations of 0.25 mM or lower were used for phenotypic endpoint assessment in this cell system.

**FIGURE 2:**
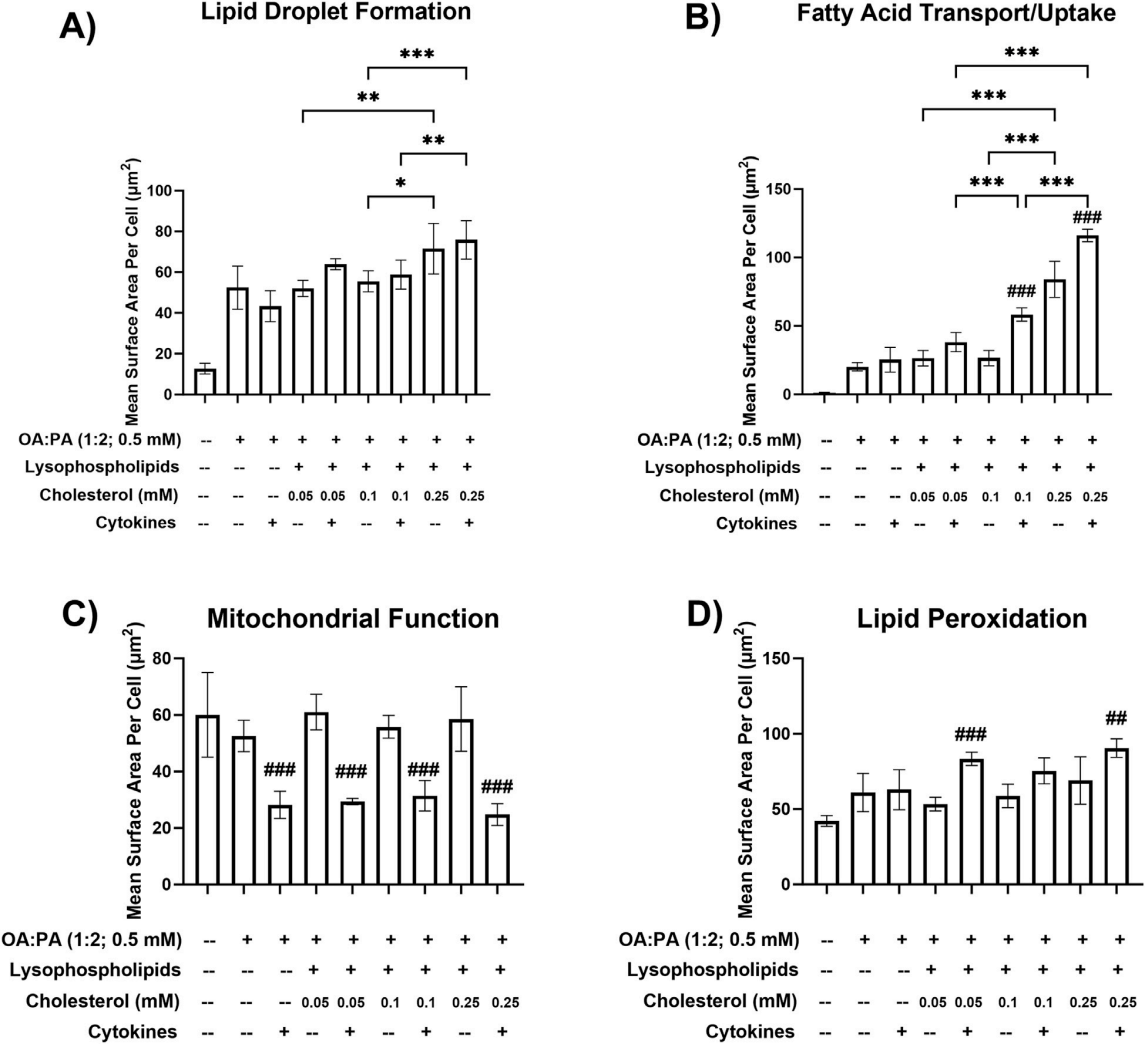
Metabolic dysfunction-associated steatohepatitis (MASH) phenotypic endpoints in a differentiated HuH-7 cell culture model following various lipid treatments with and without cytokines. Fluorescent probe-based assessment of **(A)** lipid droplet formation (BODIPY™ 493/503), **(B)** fatty acid transport/uptake (BODIPY™ 558/568 C_12_), **(C)** mitochondrial function (MitoTracker™ Orange), and **(D)** lipid peroxidation (BODIPY™ 665/676) following 72-h incubation of various lipid treatments with and without cytokines, and untreated control (0.5% DMSO), in differentiated HuH-7 cell culture using high-content imaging. A one-way analysis of variance was performed across treatments for all endpoints and corrected for multiple comparisons using Tukey’s HSD test. “*” indicates statistical significance for lipid mix-containing treatment comparisons across different cholesterol concentrations; “#” indicates statistical significance when compared to the same treatment without cytokines; *p < 0.05; **p < 0.01; ***/###p < 0.001. Six replicate measures were available for each treatment and control group (mean ± standard deviation) (see Figure 1 legend for the composition of lysophospholipid and cytokine treatments and concentrations of individual components). *DMSO, dimethyl sulfoxide; OA, oleic acid; PA, palmitic acid.*

HCI data indicated that a free cholesterol concentration of 0.25 mM enhanced intracellular lipid droplet formation and accumulation and FA transport/uptake (Figures 2A,B). Specifically, significant increases in FA uptake were observed in the lipid mix treatments containing 0.25 mM cholesterol (±cytokines) compared with treatments with 0.05 or 0.1 mM cholesterol (±cytokines). Similarly, lipid droplet formation significantly increased across these comparisons. However, no significant difference was observed between 0.25 mM cholesterol + cytokines and 0.05 mM cholesterol + cytokines. Additionally, there was a statistically significant increase in FA uptake with lipid mix + 0.1 mM cholesterol + cytokines compared with lipid mix + 0.05 mM cholesterol + cytokines. The addition of 12.5 ng/mL TNF-α and 15 ng/mL IL-6 to each lipid mix + cholesterol-containing treatment was associated with a decrease in mitochondrial function, indicating mitochondrial stress (Figure 2C). An increase in lipid peroxidation was also observed with the addition of cytokines to all treatments, except for treatment with 0.5 mM OA:PA (1:2) (Figure 2D). The observed increases were statistically significant for the 0.05 and 0.25 mM cholesterol concentrations but not for the 0.1 mM (*p* = 0.08) cholesterol concentration. Furthermore, significant increases in FA uptake were observed when cytokines were added to the lipid mix + 0.1 or 0.25 mM cholesterol treatments (Figure 2B). However, only marginal increases were noted in intracellular lipid droplet formation when cytokines were added to all treatments containing the lipid mix and cholesterol (Figure 2A). Based on promising MASH phenotype and toxicity profiles in the differentiated HuH-7 cell culture, lipid mix concentrations were kept constant while 0.05 mM–0.25 mM cholesterol, 12.5 ng/mL TNF-α, and 15 ng/mL IL-6 were selected for the initial testing in SCHH.

### 3.2 Initial assessment of toxicity associated with lipid-cytokine treatments in SCHH

**FIGURE 3:**
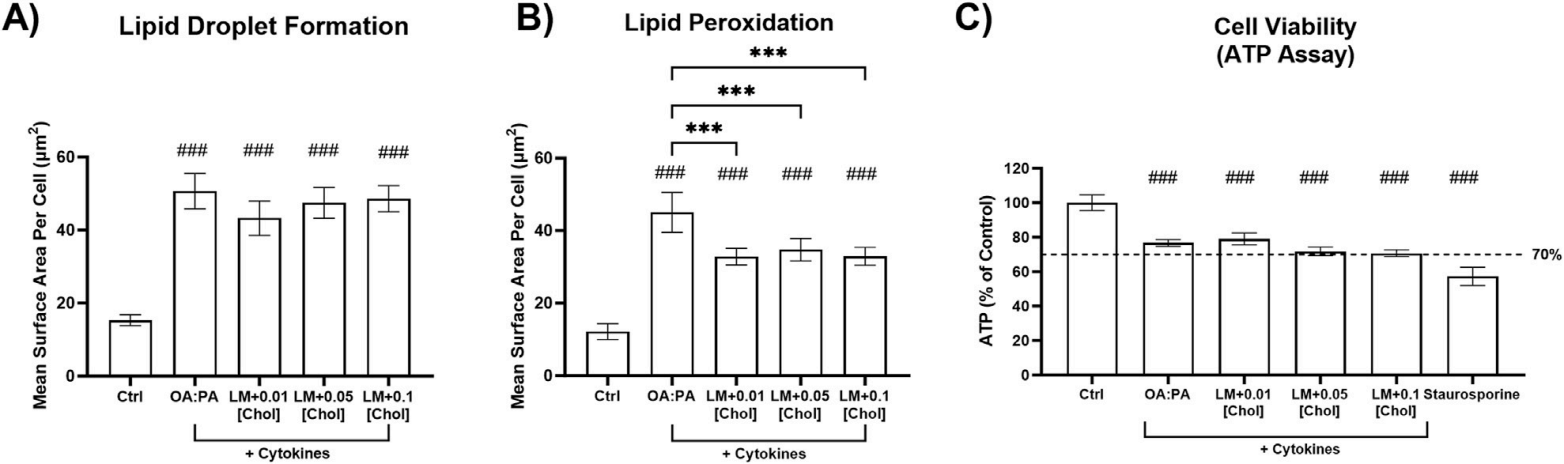
Cellular toxicity and metabolic dysfunction-associated steatohepatitis (MASH) phenotypic endpoints in a differentiated HuH-7 cell culture model after treatment with optimized lipid–cytokine treatments. Fluorescent probe-based assessment of **(A)** lipid droplet formation (BODIPY™ 493/503) and **(B)** lipid peroxidation (BODIPY™ 665/676) following 72-h incubation of various lipid treatments with and without cytokines, and untreated control (0.5% DMSO), in differentiated HuH-7 cell culture using high-content imaging. **(C)** Cell viability was assessed using the ATP assay. A pre-established “threshold” ATP luminescence value relative to the control of 70% was used to define undesirable cellular toxicity. Differentiated HuH-7 cells were treated with staurosporine (500 nM) for 24 h as a positive toxicity control. A one-way analysis of variance was performed across treatments for all endpoints and corrected for multiple comparisons using Tukey’s HSD test; “#” indicates statistical significance when compared to the vehicle control (0.5% DMSO); ***/###p < 0.001. Six replicate measures were available for each treatment and control group (mean ± standard deviation). The OA:PA treatment comprised a 2:1 ratio of OA:PA (0.5 mM total). The lipid mix (LM) comprised the OA:PA treatment + various lysophospholipids. Lysophospholipids comprised 2.5 μM 18:1 lysophosphatidylethanolamine (LysoPE) + 5 μM 18:1 lysophosphatidylcholine (LysoPC) + 1.25 μM 16:0 LysoPE +2.5 μM 16:0 LysoPC. Lysophospholipid notation “18:1” indicates a fatty acid chain of 18 carbon atoms with one double bond; “16:0” indicates a fatty acid chain of 16 carbon atoms with zero double bonds. The number preceding [chol] in the x-axis indicates the mM concentration of free cholesterol. Cytokines comprised 1.0 ng/mL TNF-α and 1.2 ng/mL IL-6. *Chol, cholesterol; DMSO, dimethyl sulfoxide; IL, interleukin; OA, oleic acid; PA, palmitic acid; TNF, tumor necrosis factor.*

During an initial assessment in SCHH (*data not shown*), undesirable cytotoxicity (relative ATP luminescence value below 70%) was observed with lipid mix + cholesterol treatments containing cytokines (12.5 ng/mL TNF-α and 15 ng/mL IL-6) or 0.25 mM cholesterol across two SCHH donors. Due to observations of cellular toxicity at higher concentrations, lower concentrations of TNF-α (1 ng/mL), IL-6 (1.2 ng/mL), and cholesterol (0.01 mM–0.1 mM) were selected for additional testing in SCHH. Lower cytokine concentrations were selected to avoid potential toxicity in SCHH while remaining within ranges that were previously observed to induce cytokine-mediated effects in primary human hepatocytes (Gramignoli et al., 2022). The toxicity and phenotype profiles of the lower cytokine and cholesterol concentrations were then assessed in differentiated HuH-7 cells before performing additional experiments in SCHH. All treatments in differentiated HuH-7 cells with the lower cytokine concentrations maintained significant increases in lipid droplet formation and lipid peroxidation (Figures 3A,B) that were also observed with higher concentrations (Figures 2A,D) and resulted in similar toxicity, as indicated by the ATP assay (Figure 3C). Ultimately, 0.01 mM cholesterol was selected for the final assessment in SCHH as this concentration appeared to have similar impact on phenotypic endpoints with a slightly lower toxicity risk than treatments containing 0.05 mM and 0.1 mM cholesterol in differentiated HuH-7 cells (Figure 3C).

### 3.3 Final assessment of toxicity and phenotypic endpoints associated with lipid-cytokine treatments in SCHH

The following three treatments were selected for the final assessment of cellular toxicity and their ability to induce MASH-like phenotypes in SCHH: 1) 0.5 mM OA:PA (1:2), 2) lipid mix, and 3) lipid mix with 0.01 mM cholesterol.

**FIGURE 4:**
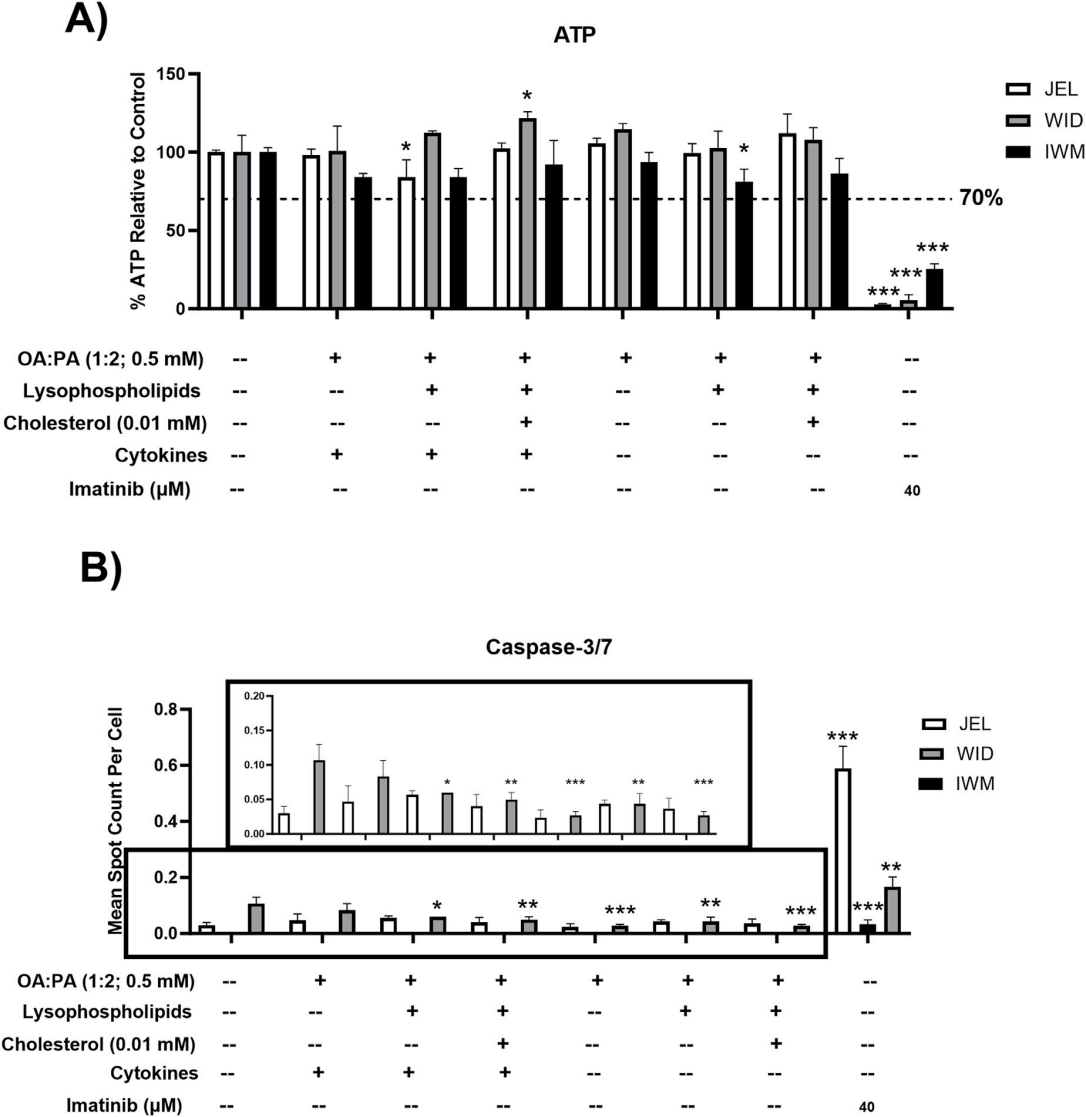
Toxicity data following 72-h exposure to 0.5% DMSO (control) or various lipid treatments with or without cytokines in sandwich-cultured human hepatocytes (SCHH) from three hepatocyte donors (JEL, WID, and IWM). **(A)** Cell viability (ATP) and **(B)** cellular apoptosis (caspase-3/7). A pre-established “threshold” ATP luminescence value relative to the untreated control (0.5% DMSO) of 70% was used to define undesirable cellular toxicity. SCHH were treated with imatinib (40 μM) for 24 h as a positive toxicity control. **(B)** Zoomed-in y-axis values from 0 to 0.2 are shown to better visualize data across treatments and the control group. Caspase-3/7 levels were undetectable in all IWM groups except for the positive toxicity control. A one-way analysis of variance was performed for both endpoints and corrected for multiple comparisons using Dunnett’s test; *p < 0.05; **p < 0.01; ***p < 0.001 vs. control. Three replicate measures per donor were available for each treatment and control group (mean ± standard deviation) (see Figure 3 legend for the composition of treatments and concentrations of components). *DMSO, dimethyl sulfoxide; OA, oleic acid; PA, palmitic acid.*

All treatments were tested for cellular toxicity with and without cytokine concentrations of 1.2 ng/mL IL-6 and 1 ng/mL TNF-α by assessing their effect on ATP and caspase-3/7 levels in three SCHH donors. Although statistically significant decreases in ATP were observed for some treatments in select donors compared with the control, none of the treatments resulted in ATP reduction below the pre-specified threshold of 70% (Figure 4A). No significant changes in caspase-3/7 activity were observed for all treatments, with and without cytokines, in SCHH donors JEL and IWM, whereas significant decreases in caspase-3/7 activity compared to control were observed in donor WID for multiple treatments with and without cytokines (Figure 4B). The mechanism underlying decreased caspase-3/7 levels in donor WID with these treatments is unknown. Compared to IWM (46 years) and JEL (27 years), WID was older (71 years) but did exhibit a lower liver fibrosis stage (F1C vs. F2) (Supplementary Table 1). Overall, minimal cellular toxicity risk was demonstrated in the three SCHH donors for all treatments with and without cytokines.

**FIGURE 5:**
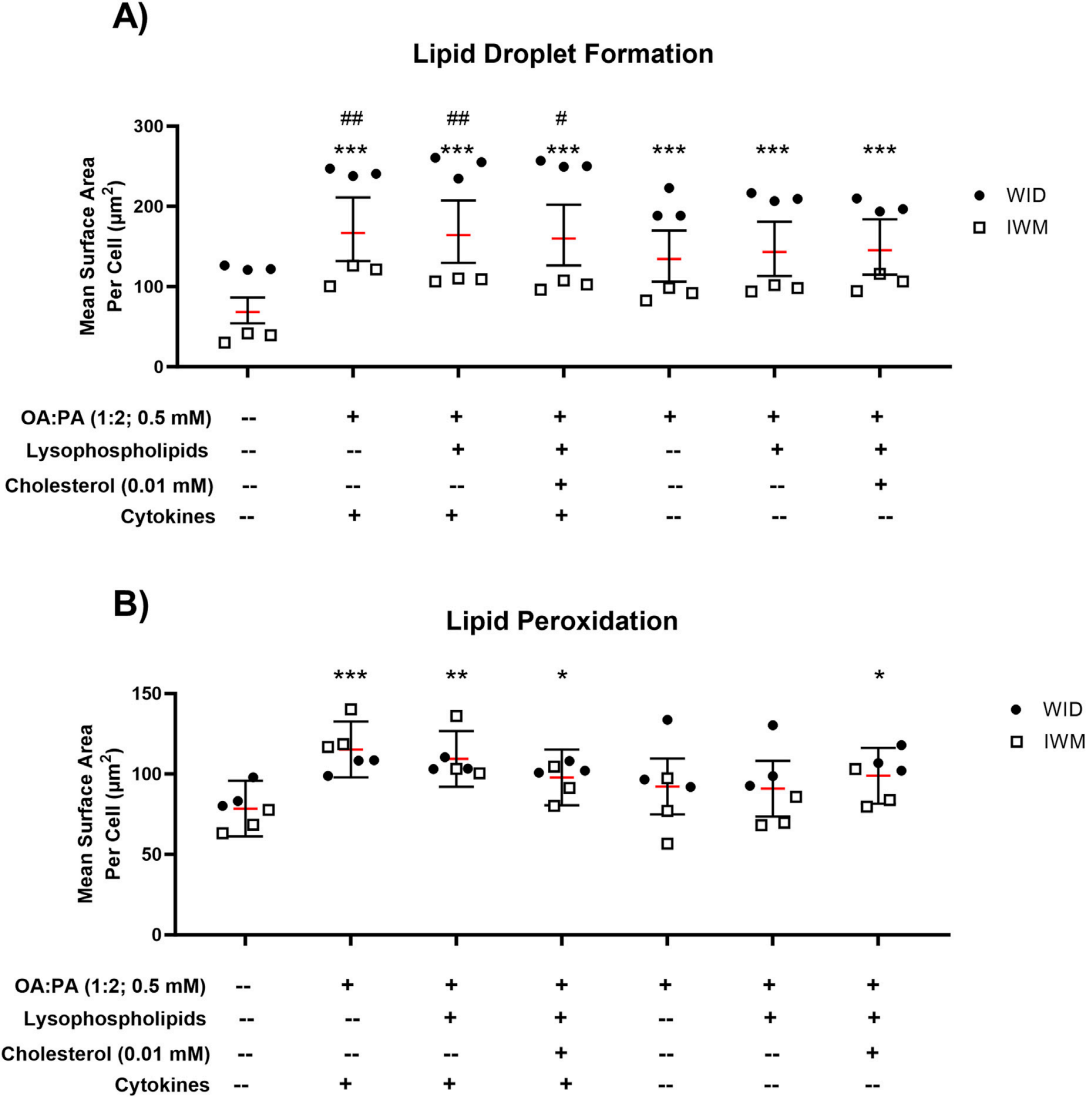
Lipid droplet formation and lipid peroxidation following 72-h exposure to 0.5% DMSO (control) or various lipid treatments with or without cytokines in sandwich-cultured human hepatocytes (SCHH) from two hepatocyte donors (WID and IWM). Fluorescent probe-based assessment of **(A)** lipid droplet formation (BODIPY™ 493/503) and **(B)** lipid peroxidation (BODIPY™ 665/676) using high-content imaging. A linear mixed-effects model was fit for each outcome with a random slope on hepatocyte donor to account for inter-donor variability. Models describing the lipid droplet formation outcome (BODIPY™ 493/503) were fit using a gamma distribution and log link function (*lme4* package (Bates et al., 2015)) in RStudio (R version 4.3). Models describing the lipid peroxidation outcome (BODIPY™ 558/568) were fit using a Gaussian distribution. When comparing treatments with and without cytokines for both outcomes, models were fit using a Gaussian distribution. “#” indicates statistical significance when compared to the same treatment without cytokines; “*” indicates statistical significance when compared to vehicle control (0.5% DMSO); */#p < 0.05; **/##p < 0.01; ***p < 0.001. Three replicate measures per donor were available for each treatment and control group (mean ± standard deviation) (see Figure 3 legend for the composition of treatments and concentrations of components). *DMSO, dimethyl sulfoxide; OA, oleic acid; PA, palmitic acid*.

To assess the ability of each treatment (with and without cytokines) to induce a MASH-like phenotype, intracellular lipid droplet formation and lipid peroxidation were assessed in two SCHH donors. Statistically significant increases in lipid droplet formation were observed for all three treatments without cytokines (Figure 5A). All treatments containing cytokines (lipid–cytokine treatments) showed statistically significant increases in lipid droplet formation compared with all treatments without cytokines. Statistically significant increases in lipid peroxidation were observed for all three treatments containing cytokines but only for one treatment without cytokines (lipid mix +0.01 mM cholesterol) (Figure 5B). Direct comparisons between the same treatments with and without cytokines revealed inconclusive differences in lipid peroxidation.

### 3.4 Quantitative targeted proteomic (QTAP) analysis of drug transport proteins

**FIGURE 6:**
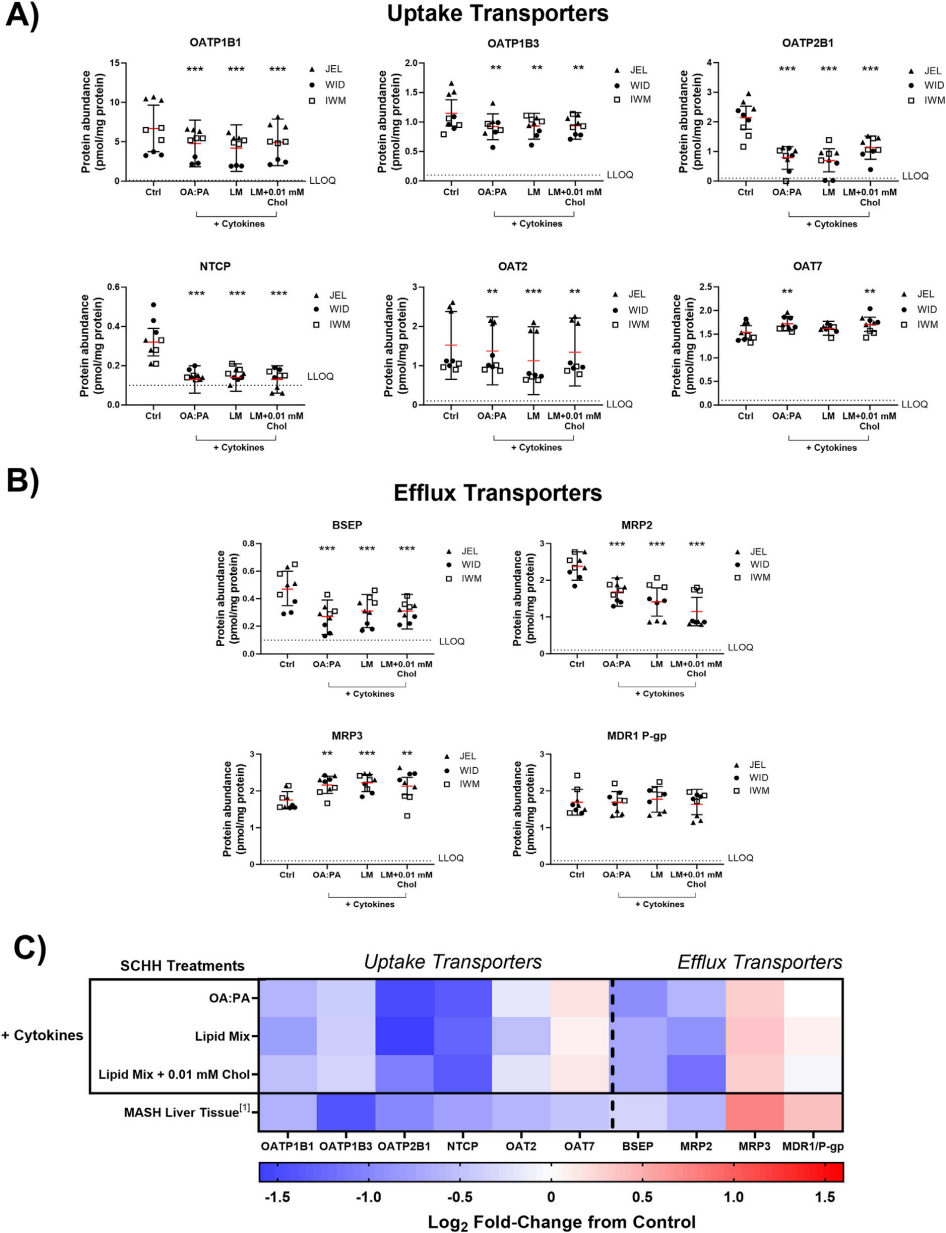
Quantitative targeted absolute proteomics (QTAP) of transporters relevant for drug disposition in sandwich-cultured human hepatocytes (SCHH) from three hepatocyte donors (JEL, WID, and IWM) following 72-h exposure to 0.5% DMSO (control) or various lipid–cytokine treatments. QTAP analysis of **(A)** uptake transporters, **(B)** efflux transporters, and **(C)** heat map comparison between QTAP SCHH data from the current study with clinically observed QTAP data in liver tissue from MASH patients (Vildhede et al., 2020)^1^. To account for treatment-specific effects on transport protein concentrations, a linear mixed-effects model was fit for each protein with a random intercept on hepatocyte donor to account for inter-donor variability. Where appropriate, models were fit using a Gaussian distribution (*lme4* package) in RStudio (R version 4.3), whereas *p* values, model predicted mean values, and 95% confidence intervals for each treatment and control group were extracted using the *lmerTest* package. The red lines **(A–B)** indicate model-predicted mean values, with the black bars indicating 95% confidence intervals; **p < 0.01; ***p < 0.001 vs. control. Using model-predicted mean values, the mean fold-change across three hepatocyte donors compared to the control was determined and converted to log_2_ fold-change for heat map comparison **(C)**. Three replicate measures per donor were available for each treatment and control group. Lipid mix (LM) comprised OA:PA + various lysophospholipids (see Figure 3 legend for more detail on treatments and components). *Chol, cholesterol; Ctrl, control; DMSO, dimethyl sulfoxide; LLOQ, lower limit of quantification; OA, oleic acid; PA, palmitic acid.*

The impact of the three selected lipid–cytokine treatments on drug transporter concentrations in SCHH from three donors (JEL, WID, and IWM) was assessed. Cytokines were included in all selected treatments due to the observed cytokine-mediated increases in SCHH lipid accumulation in donors WID and IWM (Figure 5A) and their physiologic relevance in MASH (Duan et al., 2022; Vachliotis and Polyzos, 2023). The proteomic concentrations of all basolateral uptake drug transporters of interest, except for organic anion transporter 7 (OAT7), were statistically significantly decreased across all treatments compared with untreated SCHH (control group; 0.5% DMSO) (Figure 6A). Regarding the efflux transporters (Figure 6B), BSEP and multidrug resistance-associated protein (MRP) 2 were significantly decreased while MRP3 was increased; multidrug resistance protein 1 P-glycoprotein (MDR1 P-gp) was unchanged across all treatments. Differences in transporter concentrations associated with each treatment in SCHH were converted to fold changes from control for comparison to previously published data in patients with MASH (Vildhede et al., 2020) and are depicted in Figure 6C and Supplementary Table 4. In this study, apart from OAT7, the observed changes in drug transporter concentrations were consistent in direction (i.e., increase or decrease) with previously reported alterations in liver tissue from patients with MASH (Vildhede et al., 2020).

Across all transporters, MRP2 was the only protein showing significant changes across all treatments and donors (Supplementary Figure 2). However, significant changes were observed for each transporter across all three treatments in at least two donors except for organic anion transporting polypeptide 1B3 (OATP1B3), OAT2, OAT7, and MRP3 (Supplementary Figure 2).

### 3.5 QTAP analysis of drug metabolizing enzymes (DMEs)

**FIGURE 7:**
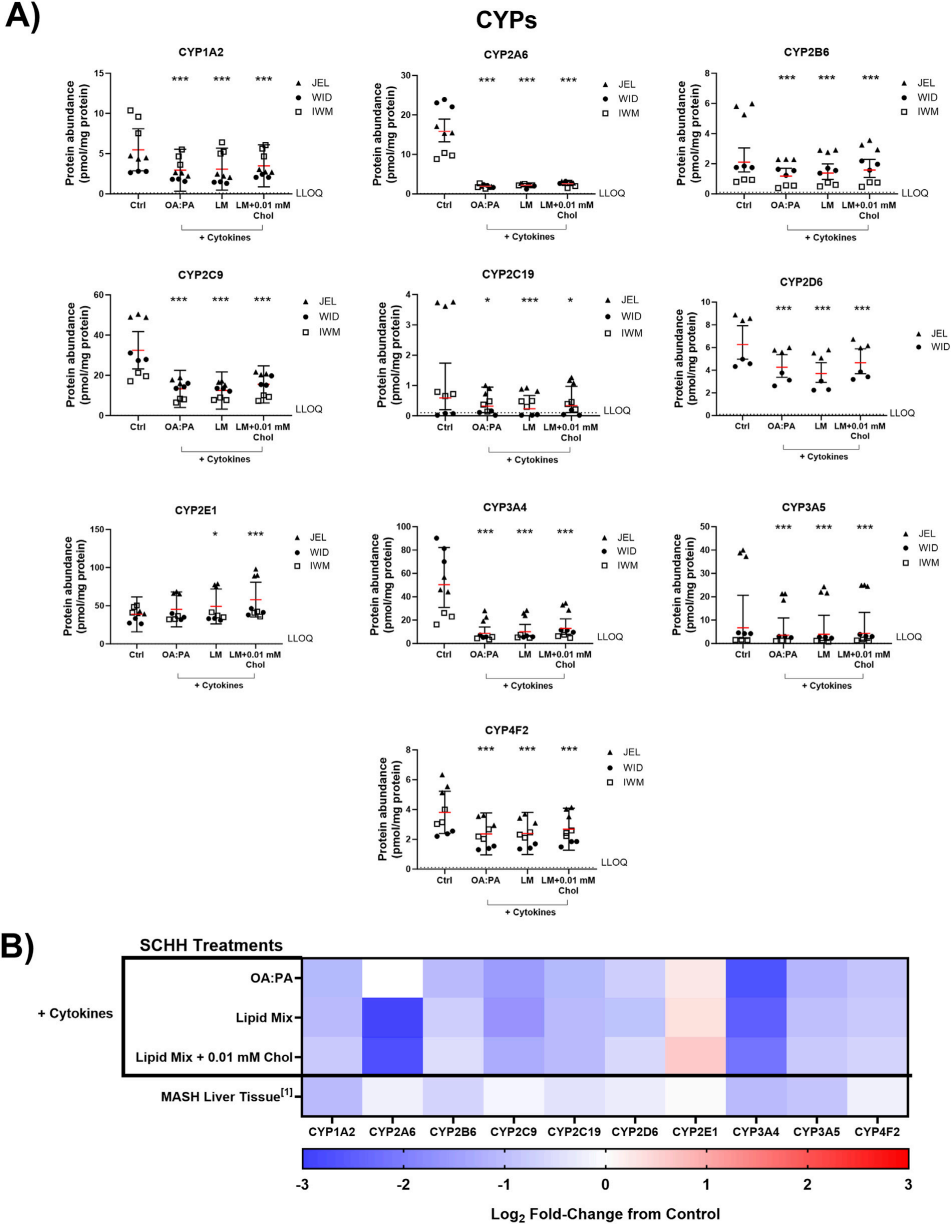
Quantitative targeted absolute proteomics (QTAP) of cytochrome P450 enzymes (CYPs) relevant for drug metabolism in sandwich-cultured human hepatocytes (SCHH) from three hepatocyte donors (JEL, WID, and IWM) following 72-h exposure to 0.5% DMSO (control) or various lipid–cytokine treatments. QTAP analysis of **(A)** CYPs and **(B)** heat map comparison between QTAP SCHH data from the current study with clinically observed QTAP data in liver tissue from MASH patients (Jamwal, 2018)^1^. To assess treatment-specific effects on CYP protein concentrations, a linear mixed-effects model was fit for each protein with a random intercept on hepatocyte donor to account for inter-donor variability. Where appropriate, models were fit using a Gaussian distribution (*lme4* package) in RStudio (R version 4.3), whereas *p* values, model predicted mean values, and 95% confidence intervals for each treatment and control group were extracted using the *lmerTest* package. For some of the analytes, the donor effects were so strong that a linear mixed-effects model created negative concentration predictions. For those models, we shifted to using a log link function for the linear mixed models, as opposed to the typical identity link function. Gaussian or gamma distributions were used for the models depending on the distribution of the raw data. The red lines **(A)** indicate model-predicted mean values, with the black bars indicating 95% confidence intervals; *p < 0.05; ***p < 0.001 vs. control. Using model-predicted mean values, the mean fold-change across three hepatocyte donors compared to the control was determined and converted to log_2_ fold-change for heat map comparison **(B)**. Three replicate measures per donor were available for each treatment and control group, except for CYP2D6, where only two donors had detectable levels of this protein across all treatments. The lipid mix (LM) comprised OA:PA + various lysophospholipids (see Figure 3 legend for more detail on treatments and components). *Chol, cholesterol; Ctrl, control; DMSO, dimethyl sulfoxide; LLOQ, lower limit of quantification; OA, oleic acid; PA, palmitic acid.*

**FIGURE 8:**
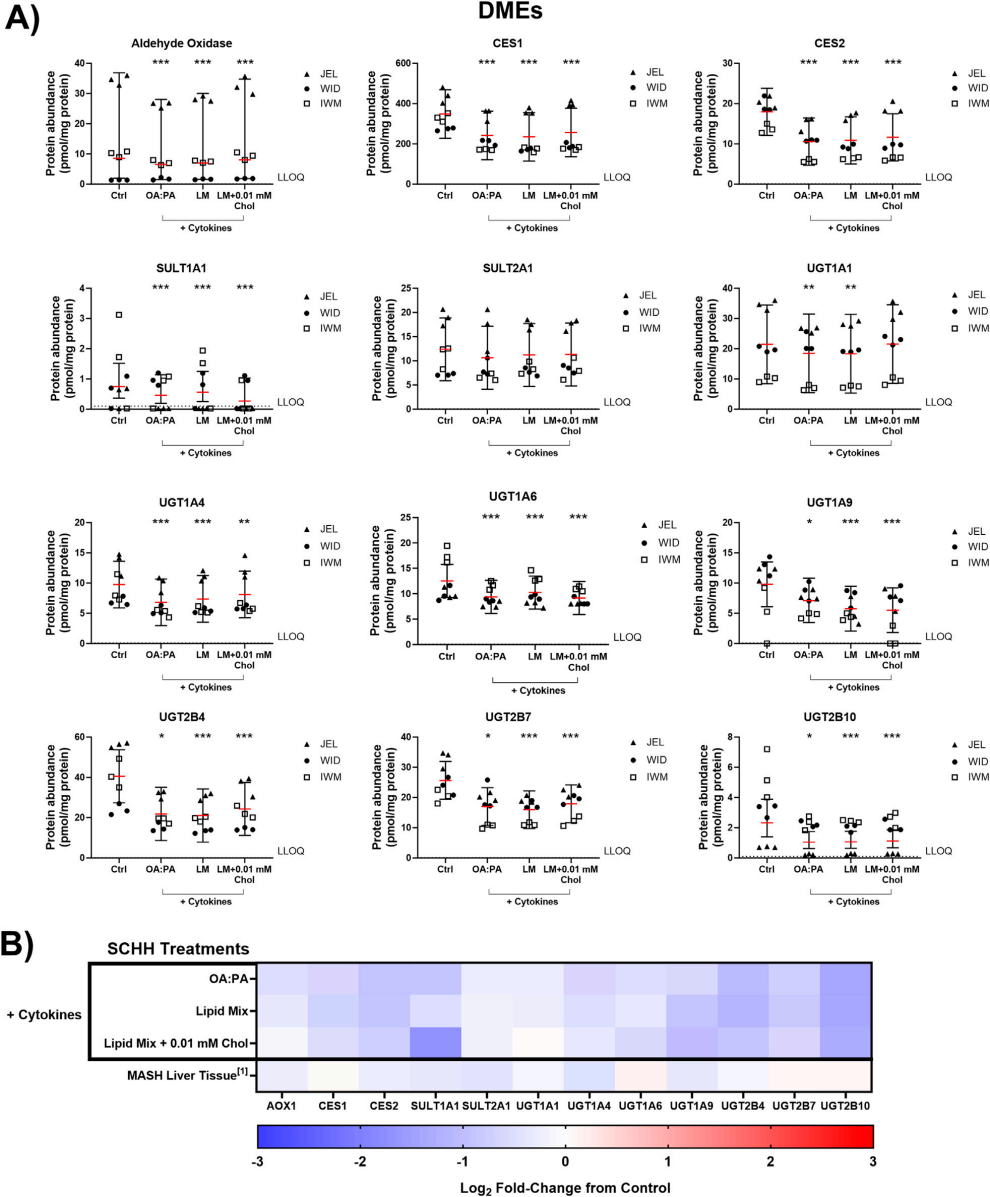
Quantitative targeted absolute proteomics (QTAP) of various non-cytochrome P450 drug metabolizing enzymes (DMEs) in sandwich-cultured human hepatocytes (SCHH) from three hepatocyte donors (JEL, WID, and IWM) following 72-h exposure to 0.5% DMSO (control) or various lipid–cytokine treatments. QTAP analysis of **(A)** DMEs and **(B)** heat map comparison between QTAP SCHH data from the current study with clinically observed QTAP data in liver tissue from MASH patients (Jamwal, 2018)^1^. To account for treatment-specific effects on DME protein concentrations, a linear mixed-effects model was fit for each protein with a random intercept on hepatocyte donor to account for inter-donor variability. Where appropriate, models were fit using a Gaussian distribution (*lme4* package) in RStudio (R version 4.3), whereas *p* values, model-predicted mean values, and 95% confidence intervals for each treatment and control group were extracted using the *lmerTest* package. For some of the analytes, the donor effects were so strong that a linear mixed-effects model created negative concentration predictions. For those models, we shifted to using a log link function for the linear mixed models, as opposed to the typical identity link function. Gaussian or gamma distributions were used for the models depending on the distribution of the raw data. The red lines **(A)** indicate model-predicted mean values, with the black bars indicating 95% confidence intervals; *p < 0.05; ***p < 0.001 vs. control. Using model-predicted mean values, the mean fold-change across three hepatocyte donors compared to the control was determined and converted to log_2_ fold-change for heat map comparison **(B)**. Three replicate measures per donor were available for each treatment and control group. The lipid mix (LM) comprised OA:PA + various lysophospholipids (see Figure 3 legend for more detail on treatments and components). *AOX1, aldehyde oxidase 1; CES, carboxylesterase; Chol, cholesterol; Ctrl, control; DMSO, dimethyl sulfoxide; LLOQ, lower limit of quantification; OA, oleic acid; PA, palmitic acid; SULT, sulfotransferase; UGT, uridine 5′-diphospho-glucuronosyltransferase.*

The impact of the three selected lipid–cytokine treatments on the proteomic concentrations of various DMEs was also assessed in SCHH from three donors (Figures 7, 8). The mean concentrations of cytochrome P450 (CYP) and uridine 5′-diphospho-glucuronosyltransferase (UGT) enzymes were significantly decreased by all treatments compared with the control, except for CYP2E1, which was increased by the lipid mix treatment with and without 0.01 mM cholesterol. Changes in UGT1A1 were inconclusive when treated with the lipid mix with 0.01 mM cholesterol. The mean concentrations of other DMEs evaluated (i.e., aldehyde oxidase 1 [AOX1], carboxylesterases [CESs], and sulfotransferases [SULTs]) were all decreased in the presence of lipid–cytokine treatments. While SULT2A1 was decreased across all treatments, no statistically significant differences were observed for this protein. Differences in DME concentrations associated with each treatment in SCHH were converted to fold changes from the control for comparison to previously published data in patients with MASH (Jamwal, 2018) and are depicted in Figures 7B, 8B. CYP3A5 concentrations were noticeably higher in JEL than in WID and IWM. This observation aligns with previous reports that CYP3A5 is expressed predominantly in hepatocyte donors of African descent (Tirona and Kim, 2017). CYP2D6 was also undetectable in all IWM samples, except for one sample treated with lipid mix + cytokines. The reason for this observation is unclear; however, it is possible that donor IWM carries a genetic variant in CYP2D6 that leads to negligible expression, given the highly polymorphic nature of this enzyme (Ingelman-Sundberg, 2005). Overall, the data indicate that changes in DME concentrations in in SCHH exposed to lipid–cytokine treatments are similar to those observed in the liver tissue from patients with MASH compared with normal liver tissue (Jamwal, 2018).

### 3.6 Functional transporter assessment using B-CLEAR^®^ technology

**FIGURE 9:**
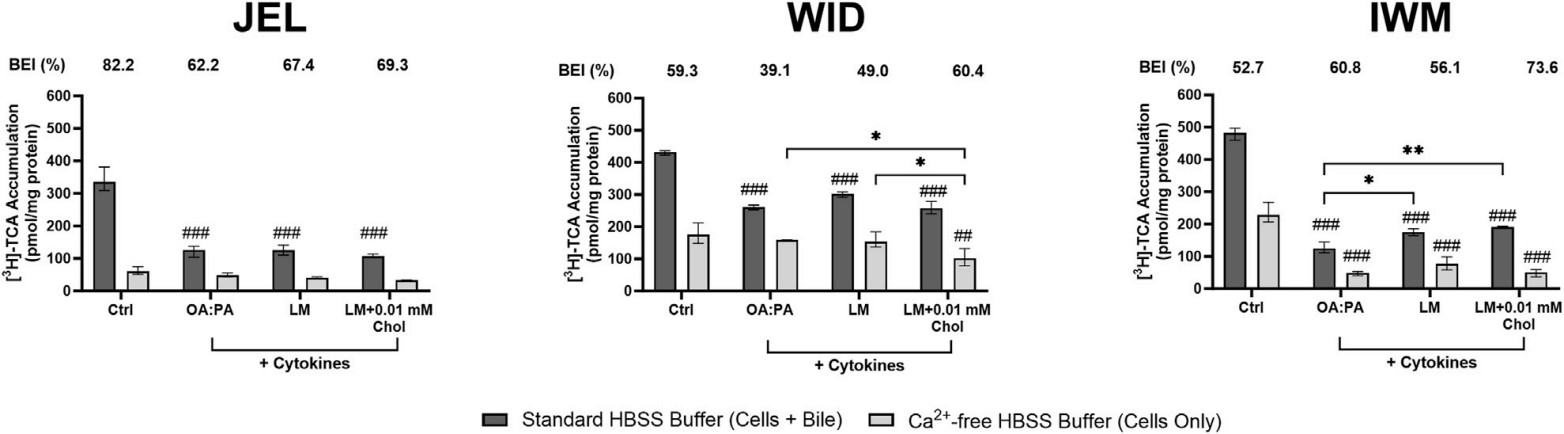
Tritiated taurocholic acid ([^3^H]-TCA) accumulation and biliary excretion index (BEI) in sandwich-cultured human hepatocytes (SCHH) from three hepatocyte donors (JEL, WID, and IWM) following 72-h exposure to 0.5% DMSO (control) or various lipid–cytokine treatments. The B-CLEAR^®^ functional assay was performed using 2 μM [^3^H]-TCA, with scintillation counting used for the quantification of cell + bile and cellular accumulation. Data are presented as the mean and standard deviation, with the calculated biliary excretion index (BEI; %) listed above each treatment or control group. Three replicate measures per donor were available for each treatment and control group. A one-way ANOVA adjusted for multiple comparisons using Tukey’s HSD test was performed across treatments for each donor; “#” indicates statistical significance when compared to the vehicle control; *p < 0.05, **/##p < 0.01, ###p < 0.001. The lipid mix (LM) comprised OA:PA + various lysophospholipids (see Figure 3 legend for more detail on treatments and components). *Chol, cholesterol; Ctrl, control; DMSO, dimethyl sulfoxide; OA, oleic acid; PA, palmitic acid*.

B-CLEAR^®^ technology was used to evaluate the hepatobiliary disposition of select transporter probe substrates in SCHH from three donors (JEL, WID, and IWM) following 72-h exposure to the three selected lipid–cytokine treatments. [^3^H]-TCA was used as a probe for sodium taurocholate co-transporting polypeptide (NTCP) and BSEP function. A significant decrease in [^3^H]-TCA “cells + bile” accumulation was observed across all donors compared with the control following exposure to each lipid–cytokine treatment (Figure 9). Accordingly, [^3^H]-TCA CL_uptake,app_ was also significantly lower than that in the control across all treatment–donor combinations (Table 2). The range of [^3^H]-TCA BEI values was 52.7%–82.2% in the control (Figure 9). Changes in BEI values varied depending on the treatment–donor combination, whereas the CL_biliary,app_ of TCA was significantly decreased by all treatments in SCHH from all donors (Table 2).

**TABLE 2:** Apparent uptake (CL_uptake,app_) and biliary clearance (CL_biliary,app_) of [^3^H]-taurocholic acid (TCA) in sandwich-cultured human hepatocytes (SCHH) from three hepatocyte donors (JEL, WID, and IWM) following 72-h exposure to 0.5% DMSO (control) or various lipid–cytokine treatments.

	TCA CL_uptake,app_ (µL/min/mg protein)				TCA CL_biliary,app_ (µL/min/mg protein)			
	DMSO control	OA:PA 1:2 (0.5 mM)	Lipid mix	Lipid mix +0.01 mM cholesterol	DMSO control	OA:PA 1:2 (0.5 mM)	Lipid mix	Lipid mix +0.01 mM cholesterol
Donor		*+ 1.0 ng/mL TNF-*α *and 1.2 ng/mL IL-6*				*+ 1.0 ng/mL TNF-*α *and 1.2 ng/mL IL-6*		
JEL	16.8 ± 1.6	6.3 ± 0.78^***^	6.3 ± 0.63^***^	5.3 ± 0.23^***^	13.8 ± 1.6	3.9 ± 0.78^***^	4.2 ± 0.63^***^	3.7 ± 0.23^***^
WID	21.6 ± 0.32	13.0 ± 0.32^***^	15.1 ± 0.37^***^	12.9 ± 0.81^***^	12.8 ± 0.32	5.1 ± 0.32^***^	7.4 ± 0.37^***^	7.8 ± 0.81^***^
IWM	24.1 ± 0.82	6.3 ± 0.73^***^	8.8 ± 0.44^***^	9.6 ± 0.07^***^	12.7 ± 0.82	3.8 ± 0.73^***^	4.9 ± 0.44^***^	7.0 ± 0.07^***^

Mean ± standard deviation of triplicate measurements. Statistical testing was performed in GraphPad Prism (version 10.1.2).

^***^p < 0.001 when compared to DMSO (control) using one-way ANOVA adjusted for multiple comparisons using Tukey’s HSD test.

Lipid mix = oleic acid (OA): palmitic acid (PA) 1:2 (0.5 mM) + 2.5 μM 18:1 LysoPE, 5 μM 18:1 LysoPC, 1.25 μM 16:0 LysoPE, 2.5 μM 16:0 LysoPC.

Abbreviations: DMSO, dimethyl sulfoxide; IL, interleukin; 16:0 LysoPC, 1-palmitoyl-2-hydroxy-sn-glycero-3-phosphocholine; 16:0 LysoPE, 1-palmitoyl-2-hydroxy-sn-glycero-3-phosphoethanolamine; 18:1 Lyso PC, 1-oleoyl-2-hydroxy-sn-glycero-3-phosphocholine; 18:1 LysoPE, 1-oleoyl-2-hydroxy-sn-glycero-3-phosphoethanolamine; TNF-α, tumor necrosis factor alpha.

**FIGURE 10:**
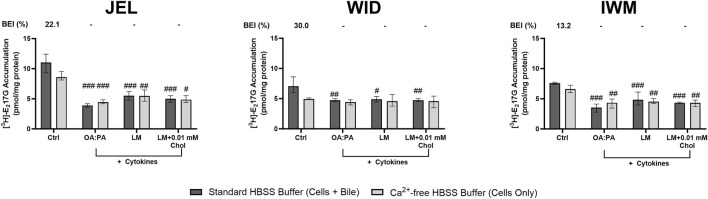
Tritiated estradiol-17β-glucuronide ([^3^H]-E_2_17G) accumulation and biliary excretion index (BEI) in sandwich-cultured human hepatocytes (SCHH) from three hepatocyte donors (JEL, WID, and IWM) following 72-h exposure to 0.5% DMSO (control) or various lipid–cytokine treatments. The B-CLEAR^®^ functional assay was performed using 0.3 μM [^3^H]-E_2_17G, with scintillation counting used for the quantification of cell + bile and cellular accumulation. Data are presented as the mean and standard deviation, with the calculated biliary excretion index (BEI; %) listed above each treatment or control group. Three replicate measures per donor were available for each treatment and control group. A one-way ANOVA adjusted for multiple comparisons using Tukey’s HSD test was performed across treatments for each donor; “#” indicates statistical significance when compared to the vehicle control; #p < 0.05, ##p < 0.01, ###p < 0.001. The lipid mix (LM) comprised OA:PA + various lysophospholipids (see Figure 3 legend for more detail on treatments and components). *Chol, cholesterol; Ctrl, control; DMSO, dimethyl sulfoxide; OA, oleic acid; PA, palmitic acid*.

The cellular disposition of [^3^H]-E_2_17G as a probe for OATP1B1, OATP1B3, and MRP2 function also was assessed in SCHH from the same three donors following exposure to the three lipid–cytokine treatments. Both the “cells + bile” accumulation and CL_uptake,app_ of [^3^H]-E_2_17G were significantly decreased across all lipid–cytokine treatments and donors (Figure 10; Table 3). BEI values for the control group ranged from 13.2% to 30%. However, BEI values were negligible across all donors for each lipid–cytokine treatment (Figure 10); thus, CL_biliary,app_ of [^3^H]-E_2_17G was not calculated.

**TABLE 3:** Apparent uptake (CL_uptake,app_) of [^3^H]-estradiol-17β-glucuronide (E_2_17G) in sandwich-cultured human hepatocytes (SCHH) from three hepatocyte donors (WID, JEL, and IWM) following 72-h exposure to 0.5% DMSO (control) or various lipid–cytokine treatments.

	E_2_17G CL_uptake,app_ (µL/min/mg protein)			
	DMSO control	OA:PA 1:2 (0.5 mM)	Lipid mix	Lipid mix +0.01 mM cholesterol
Donor		*+ 1.0 ng/mL TNF-*α *and 1.2 ng/mL IL-6*		
JEL	3.7 ± 0.43	1.3 ± 0.08^***^	1.8 ± 0.19^***^	1.7 ± 0.15^***^
WID	2.4 ± 0.37	1.6 ± 0.07^*^	1.6 ± 0.12^*^	1.6 ± 0.06^*^
IWM	2.5 ± 0.06	1.2 ± 0.19^***^	1.6 ± 0.31^*^	1.5 ± 0.03^***^

Mean ± standard deviation of triplicate measurements. Statistical testing was performed in GraphPad Prism (version 10.1.2).

^***^p < 0.001; ^*^p < 0.05 when compared to DMSO (control) using one-way ANOVA adjusted for multiple comparisons using Tukey’s HSD test. Lipid mix = oleic acid (OA): palmitic acid (PA) 1:2 (0.5 mM) + 2.5 μM 18:1 LysoPE, 5 μM 18:1 LysoPC, 1.25 μM 16:0 LysoPE, 2.5 μM 16:0 LysoPC. Abbreviations: DMSO, dimethyl sulfoxide; IL, interleukin; 16:0 LysoPC, 1-palmitoyl-2-hydroxy-sn-glycero-3-phosphocholine; 16:0 LysoPE, 1-palmitoyl-2-hydroxy-sn-glycero-3-phosphoethanolamine; 18:1 LysoPC, 1- oleoyl-2-hydroxy-sn-glycero-3-phosphocholine; 18:1 LysoPE, 1-oleoyl-2-hydroxy-sn-glycero-3-phosphoethanolamine; TNF-α, tumor necrosis factor alpha.

## 4 Discussion

MASH-mediated changes in the protein levels of hepatocyte drug transporters and DMEs are well documented (Vildhede et al., 2020; Hardwick et al., 2011; Jamwal, 2018). However, published clinical data on drug disposition in patients with MASH are limited. Importantly, *in vitro* MASH models that can be readily applied to study hepatobiliary drug transport and disposition have not been well characterized for transporters (Ramos et al., 2022; Soret et al., 2021). This has impeded understanding and accurate predictions of how observed disease-mediated transporter alterations may impact hepatobiliary drug disposition and transport function in patients with MASH. In this study, we leveraged a previously designed free FA treatment (Kralj et al., 2022) as a foundation to inform the development of an *in vitro* MASH model in SCHH to study hepatocyte drug transporters. Optimization of MASH-inducing treatments was initially performed using a cost-effective, high-throughput differentiated HuH-7 cell model (Saran et al., 2022a). This approach identified treatment concentrations that induced *in vitro* MASH-like characteristics without causing significant cellular toxicity. The impact of three MASH-inducing lipid–cytokine treatments on drug transporter and DME concentrations in SCHH was assessed and compared to previously reported observations in patients with MASH (Vildhede et al., 2020; Jamwal, 2018). Finally, the impact of lipid–cytokine treatments on transporter function was assessed using B-CLEAR^®^ technology with the probe substrate TCA for BSEP and NTCP, and E_2_17G for OATP1B1, OATP1B3, and MRP2.

In this study, the inclusion of cholesterol, TNF-α, and IL-6 with the lipid mix, which comprised free FAs and lysophospholipids, was associated with the induction of MASH-like characteristics in both differentiated HuH-7 cells and SCHH. Cellular toxicity tolerance for cytokine and cholesterol concentrations was observed to be higher in HuH-7 cells than in SCHH. This was not entirely unexpected as HuH-7 cells are a hepatocellular carcinoma-derived cell line and are likely more resistant to apoptosis and biochemical insults than primary hepatocytes (Fernald and Kurokawa, 2013). Despite some discrepancies in measured phenotype and toxicity endpoints between differentiated HuH-7 cells and SCHH, this research demonstrates that utilizing differentiated HuH-7 cells for initial MASH-inducing treatment optimization is a feasible, efficient, and economical strategy. Furthermore, these treatments were shown to induce intracellular lipid droplet formation and lipid peroxidation in SCHH, with the addition of cytokines resulting in significant increases in lipid droplet formation for two donors (Figure 5A). No particular treatment among the three lipid–cytokine treatments appeared superior in terms of inducing the MASH-like phenotype. However, based on the current understanding of MASH pathophysiology and observations of intrahepatic accumulation of free cholesterol in this disease (Puri et al., 2007), the lipid mix + 0.01 mM cholesterol lipid–cytokine treatment contains the most clinically relevant components of the disease biology.

Notably, the three selected lipid–cytokine treatments resulted in significant alterations in drug transporter concentrations that aligned with clinical observations from livers of patients with MASH, except for OAT7 (Vildhede et al., 2020). The referenced clinical study (Vildhede et al., 2020) reported inconclusive decreases in BSEP, MRP2, and OAT7 levels. This *in vitro* study is also the first known study to directly evaluate the effects of lipid and/or cytokine exposure on OAT7. It is possible that the clinical study (Vildhede et al., 2020) was statistically underpowered to detect true changes in OAT7 concentrations, unlike the current *in vitro* study, which also observed significant decreases in BSEP and MRP2 following lipid–cytokine treatment exposure. Notably, BSEP abundance was recently shown to be decreased in pericentral regions of the liver acinus from patients with MASH (Murphy et al., 2024). The pericentral region is typically the main site of steatosis and inflammation in adult patients with MASH (Steinman et al., 2021). The addition of TNF-α and IL-6 to human hepatocytes has been shown to decrease BSEP abundance while inducing some characteristics of hepatic inflammation/injury *in vitro* (Gramignoli et al., 2022), suggesting a potential role for these mechanisms in BSEP downregulation. Considering that the lipid–cytokine treatments used in this study contained TNF-α and IL-6 concentrations similar to those reported in the previous study, the observed decrease in BSEP concentrations are not surprising. Furthermore, despite previous observations of inconclusive decreases in hepatic MRP2 concentrations (Vildhede et al., 2020), MRP2 protein has been reported to be less glycosylated and localized intracellularly rather than on the plasma membrane in liver tissue from patients with MASH (Hardwick et al., 2011; Canet et al., 2015). This finding is supported by observations of impaired MRP2 function in patients with MASH (Ali et al., 2018).

Most transporters did not differ across treatments. However, MRP2 concentrations were reduced more with the lipid mix and lipid mix + 0.01 mM cholesterol + cytokine treatments than with the 0.5 mM OA:PA (1:2) + cytokine treatment. Additionally, NTCP concentrations appeared to exhibit the largest decrease with the lipid mix + 0.01 mM cholesterol treatment (Supplementary Table 4). The underlying mechanism(s) behind these varied responses remain unclear. However, the pronounced decrease in NTCP concentrations with the addition of cholesterol, a key precursor for bile acid synthesis, may be attributed to NTCP downregulation mediated by the farnesoid X receptor. A previous study reported that NTCP mRNA levels decreased by approximately two-fold in one SCHH donor after 72 h of exposure to chenodeoxycholic acid, whereas two other SCHH donors showed minor increases of less than two-fold (Jackson et al., 2016). Although BSEP, another farnesoid X receptor-regulated transporter (Ananthanarayanan et al., 2001), did not show a similar pattern, this may be due to its longer half-life (∼4–6 days (Soroka and Boyer, 2014; Kipp et al., 2001)) than NTCP (<24 h (Tsukuda et al., 2015)). This effect may additionally be confounded by the presence of cytokines, which can decrease BSEP abundance/function in human hepatocytes (Gramignoli et al., 2022; Diao et al., 2010). In summary, all three lipid–cytokine treatments under investigation effectively induced MASH-like changes in SCHH transporter concentrations. The lipid mix + 0.01 mM cholesterol + cytokine treatment, which led to the most substantial decrease in MRP2 (Supplementary Table 4), is particularly noteworthy due to clinical evidence of reduced MRP2 function in patients with MASH (Ali et al., 2018).

The effects of the lipid–cytokine treatments on BSEP, MRP2, NTCP, OATP1B1, and OATP1B3 were further evaluated using the probe substrates TCA and E_2_17G to assess the transporter function. Decreases in [^3^H]-TCA accumulation, uptake, and biliary clearance were observed across the three SCHH donors, which may reflect observations of reduced protein concentrations of NTCP and BSEP in this model. Observations of increased total bile acid concentrations in the serum and increased glycocholate and TCA concentrations in urine have been described clinically in MASH (Ferslew et al., 2015). Reduced function of these transporters may have clinical implications for the disposition of drugs transported by NTCP (e.g., statins) and could increase the risk of drug-induced liver injury from medications that inhibit BSEP in the presence of MASH. Prior literature have proposed an elevated risk of drug-induced liver injury in patients with MASH (Mosedale and Watkins, 2020; Lammert et al., 2019; Ghabril et al., 2025), and the current findings may provide additional mechanistic support for this hypothesis. The observed decrease in [^3^H]-E_2_17G uptake clearance across all three donors is consistent with the observed reductions in protein concentrations of OATP1B1, OATP1B3, and MRP2. As noted above, reduced MRP2 function has been reported in clinical studies in patients with MASH. The functional activity of OATP1B1 and OATP1B3 in MASH has also been examined clinically although results have been mixed. Two studies evaluating the disposition of silymarin and its metabolites observed more than two-fold increases in plasma AUC in MASLD patients (Schrieber et al., 2011; Schrieber et al., 2008); however, these compounds are also substrates for MRP2, which may confound interpretation. In contrast, a separate study assessing the pharmacokinetics of rosuvastatin found no significant differences between MASH patients and healthy controls (Tirona et al., 2018). Interpretation of this result is complicated by the involvement of additional hepatic transporters in rosuvastatin disposition (e.g., MRP2, MRP3, and BCRP), potentially masking changes in OATP1B1 and OATP1B3 activity. Moreover, the Tirona et al. (2018) study enrolled a heterogeneous cohort of 22 MASLD subjects, including individuals with both MASL and MASH; this study may have been underpowered to detect true differences due to high inter-individual variability in rosuvastatin pharmacokinetics (Courlet et al., 2021). In another clinical study (Ali et al., 2018), patients with biopsy-confirmed MASH exhibited increased systemic and hepatic exposure to ^99m^Tc-mebrofenin, along with a more than 2-fold reduction in biliary clearance than age- and sex-matched healthy subjects. These alterations were consistent with MASH-associated decreases in hepatic uptake (*via* OATP1B1 and OATP1B3) and biliary clearance (*via* MRP2). Findings from the current study support reduced function of OATP1B1, OATP1B3, and MRP2 in MASH, highlighting the potential for altered disposition of drugs transported by these pathways in MASH patients. Although MRP4 has been reported to be upregulated in MASH (Hardwick et al., 2011), it was below LLOQ in most samples in our study, which is consistent with previous QTAP-based analyses in liver tissue (Vildhede et al., 2020) and SCHH (Fashe et al., 2022).

The three lipid–cytokine treatments also induced changes in various DMEs that were consistent with those previously reported in livers from MASH patients (Jamwal, 2018). Although functional assessment of DME activity was not performed in SCHH following lipid–cytokine exposure, changes in DME protein levels are generally known to correlate well with functional activity (Ohtsuki et al., 2012). Supporting this, several clinical studies in MASLD patients have demonstrated concordance between alterations in CYP enzyme abundance and function (Li et al., 2017; Woolsey et al., 2015; Kawaguchi-Suzuki et al., 2017; Manitpisitkul et al., 2014).

Pro-inflammatory cytokines were necessary to be included in the *in vitro* MASH model to accurately reflect clinical MASH pathophysiology (Duan et al., 2022; Vachliotis and Polyzos, 2023). Further investigations would be required to determine whether observed changes in transport protein and DME concentrations are driven predominantly by cytokine or lipid exposure, or a combination of both. Previous research that examined the effects of cytokines on various hepatocyte transporters demonstrated reduced mRNA, protein abundance, and/or activity of OATP1B1, OATP1B3, OATP2B1, NTCP (Le Vee et al., 2009; Hao et al., 2024), MRP2 (Diao et al., 2010; Le Vee et al., 2009), and BSEP (Gramignoli et al., 2022; Diao et al., 2010), following exposure to TNF-α and/or IL-6. MRP3 demonstrated increased protein abundance following TNF-α and IL-6 exposure in human hepatocytes (Le Vee et al., 2009). It is well established that TNF-α and IL-6 decrease CYP abundance and function (Frye et al., 2002; Yu et al., 2023). The association between TNF-α and IL-6 on the function of other DMEs is less well established. Decreased UGT1A1 function in primary human hepatocytes was also observed following exposure to TNF-α and IL-6 (Gramignoli et al., 2022). Decreased UGT1A4, UGT2B4, and UGT2B7 mRNA levels were associated with high inflammation scores in human liver biopsies (Congiu et al., 2002). These data suggest that observed changes in transporter and DME concentrations or function may be largely driven by cytokines. Confirming this would require testing the effects of lipid and cytokine components on transporter concentrations in this model separately. These studies were beyond the scope of the current project.

Although cytokines alongside FAs and other lipids have been added to primary human hepatocyte-derived cultures to induce a MASH-like phenotype previously (Ströbel et al., 2021), this study is the first to evaluate the impact of cytokines combined with various lipid components pertinent to MASLD pathogenesis on drug transporters and DMEs in primary human hepatocytes cultured in a sandwich configuration. More complex MASH models that incorporate non-parenchymal cells involved in MASLD progression and liver injury (Kumar et al., 2021), or that allow for longer term studies, may offer a more physiologically relevant *in vitro* environment to predict altered drug disposition in patients with MASH. Nevertheless, the optimized lipid–cytokine treatment conditions in this study effectively induced key aspects of the MASH phenotype in SCHH (lipid droplet formation and lipid peroxidation) and induced clinical MASH-like alterations in the concentrations of drug transport proteins and DMEs (Vildhede et al., 2020; Jamwal, 2018). Similar impacts on transporter function have been observed in clinical studies (Ali et al., 2018; Schrieber et al., 2008).

In summary, the three identified lipid–cytokine treatments induced significant lipid droplet formation and lipid peroxidation in SCHH, along with changes in the concentrations and/or function of transporters and DMEs that mirror those observed in patients with MASH. These treatments hold potential for future applications in SCHH models to better predict altered hepatobiliary drug disposition and to support *in vitro*-to-*in vivo* extrapolation (IVIVE) approaches for pharmacokinetic modeling in the MASH population. As a future direction, comprehensive lipidomic profiling of SCHH treated with each lipid–cytokine combination will be performed to identify the treatment that most closely replicates the intrahepatic lipidome observed in clinical MASH.

## Data Availability

The mass spectrometry proteomics data have been deposited to the ProteomeXchange Consortium via the PRIDE (Perez-Riverol et al., 2022) partner repository with the dataset identifier PXD071083. Available at: https://www.ebi.ac.uk/pride/archive/projects/PXD071083.
