# Supplementary material for: Development of an in vitro metabolic dysfunction-associated steatohepatitis model to investigate altered drug metabolizing enzymes, transport proteins, and hepatobiliary disposition
Source: Front Pharmacol. 2026 Jan 12;16:1664808. doi: 10.3389/fphar.2025.1664808 (PMC12832903; doi:10.3389/fphar.2025.1664808)
Supplement: Supplementary file 1 [file DataSheet1.pdf]

Supplementary Material

Development of an *In Vitro* Metabolic Dysfunction-Associated Steatohepatitis Model to Investigate Altered Drug Metabolizing Enzymes, Transport Proteins, and Hepatobiliary Disposition

William A. Murphy[1], Sarina Kyburz[1,2], Henry Ho[1], Matthew Shane Loop[1,3], John K. Fallon[4], Jacqueline B. Tiley[1], Thomas Kralj[5], Kim L.R. Brouwer[1*]

[1]Division of Pharmacotherapy and Experimental Therapeutics, UNC Eshelman School of Pharmacy, University of North Carolina at Chapel Hill, Chapel Hill, North Carolina, USA

[2]Biopharmacy, Department of Pharmaceutical Sciences, University of Basel, Basel, Switzerland

[3]Department of Health Outcomes Research and Policy, Harrison College of Pharmacy, Auburn University, Auburn, Alabama, USA

[4]Division of Pharmacoengineering and Molecular Pharmaceutics, and Center for Nanotechnology in Drug Delivery, UNC Eshelman School of Pharmacy, University of North Carolina at Chapel Hill, Chapel Hill, NC, USA

[5]Pharmaron Lab Services Inc., Germantown, MD, USA

***Correspondence:**

Kim L.R. Brouwer, PharmD, PhD
Address: Division of Pharmacotherapy and Experimental Therapeutics,
UNC Eshelman School of Pharmacy, University of North Carolina at Chapel Hill, CB #7569
Kerr Hall, Chapel Hill, NC 27599-7569. Telephone: (919) 962-7030. Fax: (919) 962-0644.
Email: kbrouwer@unc.edu

1 Supplementary Figures and Tables

1.1 Supplementary Figures

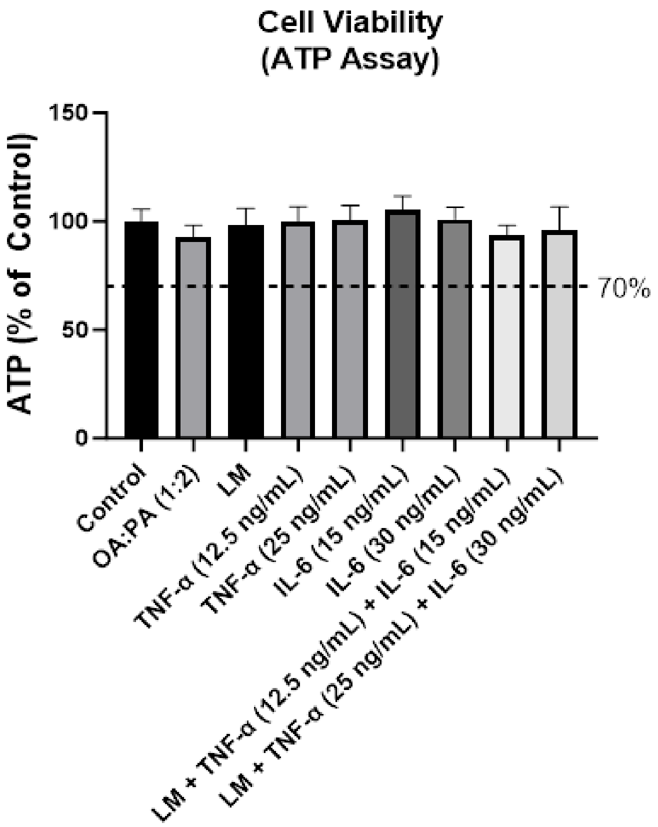

Supplementary Figure 1. Effect of Cytokines on Cell Viability in Differentiated HuH-7 cells. Higher cytokine concentrations (TNF-α, 25 ng/mL; IL-6, 30 ng/mL) independently and in combination with the Lipid Mix (LM) showed no notable toxicity in differentiated HuH-7 cell culture following a 72-hour exposure using the ATP assay. A pre-established "threshold" ATP luminescence value relative to control of 70% was used to define undesirable cellular toxicity. Mean ± standard deviations are shown. A one-way analysis of variance was performed and corrected for multiple comparisons to control using Dunnett's test; no statistically significant differences were observed for any treatment group compared to control. At least 12 replicate measurements were available for each treatment group. The Lipid Mix was comprised of oleic acid (OA; 167 µM) and palmitic acid (PA; 333 µM) and various lysophospholipids (11.25 µM total). *IL, interleukin; TNF, tumor necrosis factor.*

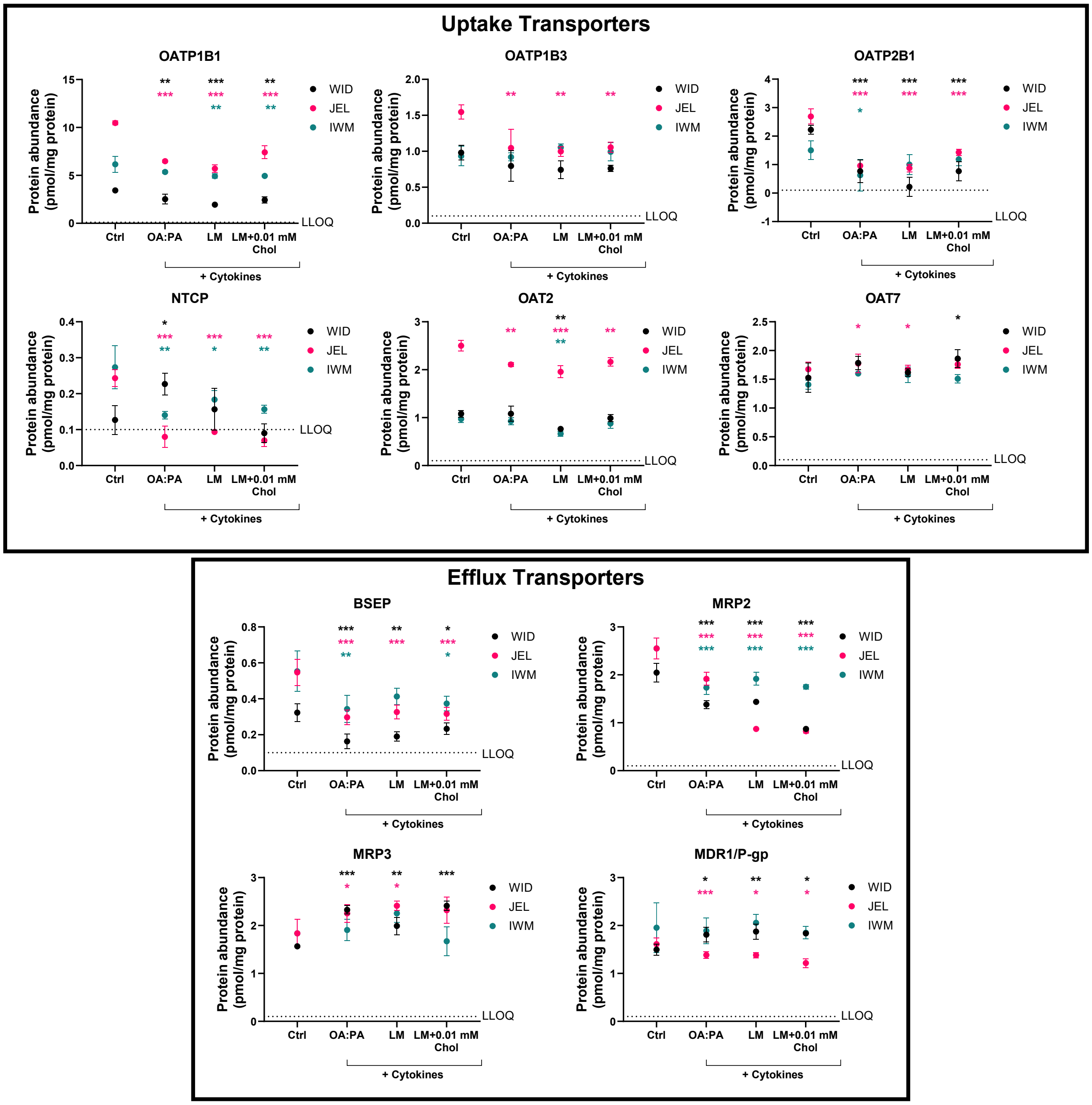

Supplementary Figure 2. Quantitative targeted absolute proteomics (QTAP) analysis of transport proteins analyzed separately by hepatocyte lot. Means ± standard deviations are shown. Instances where error bars are not visible indicate that the standard deviation range is smaller than the visual size of the data point itself. A linear regression model was fit separately for each hepatocyte lot and transport protein combination using the *lm* function using R version 4.3 (*stats* package); all treatments were compared to the control (Ctrl) group. **p*<0.05; ***p*<0.01; ****p*<0.001 (color coding represents the significance value for the corresponding lot shown in the figure key). The Lipid Mix was comprised of oleic acid (OA; 167 µM) and palmitic acid (PA; 333 µM) and various lysophospholipids (11.25 µM total). SCHH were exposed to all treatments for 72 hours. *BSEP, bile salt export pump; Chol, cholesterol; Ctrl, Control; LLOQ, lower limit of quantification; LM, lipid mix; MDR1/P-gp, multidrug resistance protein 1/P-glycoprotein; MRP, multidrug resistance–associated protein; NTCP, sodium-taurocholate cotransporting polypeptide; OA, oleic acid; OAT,*

organic anion transporter; OATP, organic anion transporting polypeptide; PA, palmitic acid; P-gp, p-glycoprotein

1.2 Supplementary Tables

Supplementary Table 1. Cryopreserved Transporter Certified™ human hepatocyte donor demographics.

	Donor		
**Demographics**	**WID**	**JEL**	**IWM**
Age (years)	71	27	46
Gender (M/F)	M	F	M
Race	White	African American	White
BMI (kg/m^2)	25.7	28.2	23.4
COD	Head Trauma/Blunt Injury/MVA	Anoxia	Anoxia
Medical History	Hypertension x 15 yrs	Respiratory disease & quadriplegic	Asthma, cystic fibrosis, hypertension
Medication/Drug History	None Reported	None Reported	Crack cocaine, THC, meth
Social History	None reported	None reported	Crack cocaine, marijuana, methamphetamine use
NAS Score	5	3	6
Fibrosis Stage	1C	2	2

BMI, body-mass index; COD, cause of death; F, female; M, male; MVA, motor vehicle accident; NAS, NAFLD Activity Score; THC, tetrahydrocannabinol.

Supplementary Table 2. Fluorescent probe concentrations/incubation times and high-content imaging (HCI) parameters using the CellInsight™ CX7 LZR High Content Analysis platform.

Probe	Detection/Measurement Target	Concentration	Incubation Time (min)	Emission Wavelength (nm)	HCI Excitation Laser Wavelength (nm)	HCI Capture Settings (Dichroic Mirror_Emission Filter)
BODIPY™ 493/503	Neutral Lipids	5 µM	30	490-501	488	BGS_BGS
BODIPY™ 558/568 C12	Fatty Acid Uptake	1 µM	~24 hr	555-566	561	BGRFr_RS
BODIPY™ 665/676	Lipid Peroxidation	10 µM	30	~676	647	BGRFr_BGRFr
MitoTracker™ Orange	Mitochondrial potential	50 nM	30	550-600	561	BGRFr_RS
Hoechst Blue 33342	Cell Nuclei	3.3 µM	30-45	460-490	405	BGRFr_BGRFr
CellEvent™ Caspase-3/7	Caspase 3/7 (Apoptotic Marker)	5 µM	45	519-575	488	BGS_BGS

Detection Reagent						

B, blue; Fr, far red; G, green; HCI, high-content imaging; hr, hours; min, minutes; R, red; S, scarlet.

Supplementary Table 3. Tryptic proteotypic human peptides used to report transporter and drug metabolizing enzyme protein concentrations in sandwich-cultured human hepatocytes that were above the lower limit of quantification of 0.1 pmol/mg protein, and multiple-reaction monitoring (MRM) transitions (*i.e.*, MRM1, MRM2) employed for each (unlabeled = Analyte; SIL [stable isotope labeled] = Internal Standard).

**Protein (*Gene Name*)**	**Peptide Sequence**	**Analyte MRM1, MRM2 (precursor ion/product ion)**	**Internal Standard MRM1, MRM2 (precursor ion/product ion)**
BSEP (*ABCB11*)	TVQVALD**K**[a]	437.26/673.39 (y6), 437.26/545.33 (y5)	441.26/681.40 (y6), 441.26/553.34 (y5)
MDR1 P-gp (*ABCB1*)	IIDNKPSIDSYS**K**	493.93/627.30 (y11), 493.93/896.44 (y8)	496.60/631.31 (y11), 496.60/904.45 (y8)
MRP2 (*ABCC2*)	LTIIPQDPILFSGSL**R**	885.51/441.31 (b4), 885.51/989.58 (y9)	890.52/441.31 (b4), 890.52/999.59 (y9)
MRP3 (*ABCC3*)	GALVAVVGPVGCG**K**	642.36/674.33 (y7), 642.36/773.40 (y8)	646.37/682.34 (y7), 646.37/781.41 (y8)
NTCP (*SLC10A1*)	GIYDGDL**K**	440.72/710.34 (y6), 440.72/547.27 (y5)	444.73/718.35 (y6), 444.73/555.29 (y5)
OAT2 (*SLC22A7*)	NVALLALP**R**	483.81/753.50 (y7), 483.81/569.38 (y5)	488.81/763.51 (y7), 488.81/579.39 (y5)
OAT7 (*SLC22A9*)	DTLTLEIL**K**	523.31/716.46 (y6), 523.31/829.54 (y7)	527.32/724.47 (y6), 527.32/837.55 (y7)
OATP1B1 (*SLCO1B1*)	NVTGFFQSF**K**	587.80/961.48 (y8), 587.80/860.43 (y7)	591.81/969.49 (y8), 591.81/868.44 (y7)

OATP1B3 (*SLCO1B3*)	IYNSVFFG**R**	551.79/826.42 (y7), 551.79/712.38 (y6)	556.79/836.43 (y7), 556.79/722.39 (y6)
OATP2B1 (*SLCO2B1*)	YYNNDLL**R**	535.77/744.40 (y6), 535.77/907.46 (y7)	540.77/754.41 (y6), 540.77/917.47 (y7)
CYP1A2 (*CYP1A2*)	YLPNPALQ**R**	536.30/398.23 (y7), 536.30/584.35 (y5)	541.31/403.23 (y7), 541.31/594.36 (y5)
CYP2A6 (*CYP2A6*)	GTGGANIDPTFFLS**R**	776.89/982.50 (y8), 776.89/867.47 (y7)	781.90/992.51 (y8), 781.90/877.48 (y7)
CYP2B6 (*CYP2B6*)	GYIIP**K**	345.71/470.33 (y4), 345.71/357.25 (y3)	349.73/478.36 (y4), 349.73/365.27 (y3)
CYP2C9 (*CYP2C9*)	SLVDP**K**	329.69/458.26 (y4), 329.69/244.17 (y2)	333.70/466.28 (y4), 333.70/252.18 (y2)
CYP2C19 (*CYP2C19*)	GHFPLAE**R**	309.50/439.21 (b4), 309.50/375.20 (y3)	312.84/439.21 (b4), 312.84/385.21 (y3)
CYP2D6 (*CYP2D6*)	SQGVFLA**R**	439.25/662.40 (y6), 439.25/506.31 (y4)	444.25/672.41 (y6), 444.25/516.32 (y4)
CYP2E1 (*CYP2E1*)	FITLVPSNLPHEAT**R**	565.65/561.29 (y10), 565.65/710.36 (y6)	568.98/566.29 (y10), 568.98/720.37 (y6)
CYP3A4 (*CYP3A4*)	LSLGGLLQPEKPVVL**K**	564.35/689.43 (y13), 564.35/660.92 (y12)	567.03/693.43 (y13), 567.03/664.92 (y12)
CYP3A5 (*CYP3A5*)	DTINFLS**K**	469.25/721.42 (y6), 469.25/217.08 (b2)	473.26/729.44 (y6), 473.26/217.08 (b2)

CYP4F2 (*CYP4F2*)	SVINASAAIAP**K**	437.26/673.39 (y6), 437.26/545.33 (y5)	441.26/681.40 (y6), 441.26/553.34 (y5)
Aldehyde Oxidase (*AOX1*)	HLGTLAGSQI**R**	493.93/627.30 (y11), 493.93/896.44 (y8)	496.60/631.31 (y11), 496.60/904.45 (y8)
CES1 (*CES1*)	ELIPEATE**K**	885.51/441.31 (b4), 885.51/989.58 (y9)	890.52/441.31 (b4), 890.52/999.59 (y9)
CES2 (*CES2*)	LGVLGFFSTGD**K**	642.36/674.33 (y7), 642.36/773.40 (y8)	646.37/682.34 (y7), 646.37/781.41 (y8)
SULT1A1 (*SULT1A1*)	VHPEPGTWDSFLE**K**	440.72/710.34 (y6), 440.72/547.27 (y5)	444.73/718.35 (y6), 444.73/555.29 (y5)
SULT2A1 (*SULT2A1*)	TLEPEELNLIL**K**	483.81/753.50 (y7), 483.81/569.38 (y5)	488.81/763.51 (y7), 488.81/579.39 (y5)
UGT1A1 (*UGT1A1*)	DGA**F**YTLK	523.31/716.46 (y6), 523.31/829.54 (y7)	527.32/724.47 (y6), 527.32/837.55 (y7)
UGT1A4 (*UGT1A4*)	VTLGYTQG**F**FETEHLLK	587.80/961.48 (y8), 587.80/860.43 (y7)	591.81/969.49 (y8), 591.81/868.44 (y7)
UGT1A6 (*UGT1A6*)	DIVEVLSD**R**	551.79/826.42 (y7), 551.79/712.38 (y6)	556.79/836.43 (y7), 556.79/722.39 (y6)
UGT1A9 (*UGT1A9*)	GILCHYLEEGAQCPAPLSYVP**R**	535.77/744.40 (y6), 535.77/907.46 (y7)	540.77/754.41 (y6), 540.77/917.47 (y7)

UGT2B4 (*UGT2B4*)	FSPGYAIE**K**	536.30/398.23 (y7), 536.30/584.35 (y5)	541.31/403.23 (y7), 541.31/594.36 (y5)
UGT2B7 (*UGT2B7*)	ADVWLI**R**	776.89/982.50 (y8), 776.89/867.47 (y7)	781.90/992.51 (y8), 781.90/877.48 (y7)
UGT2B10 (*UGT2B10*)	GHEVTVLASSASILFDPNDSSTL**K**	345.71/470.33 (y4), 345.71/357.25 (y3)	349.73/478.36 (y4), 349.73/365.27 (y3)

[a]Only one MRM (unlabeled and SIL) was detected for some samples from donors JEL and WID.
Bold letters denote amino acids labeled with a stable isotope (^{13}C and ^{15}N). Trypsin is not believed to cleave the amide bond between lysine (K) and proline (P) highlighted in red (KP). *BSEP, bile salt export pump; CES, carboxylesterase; CYP, cytochrome P450; MDR1 P-gp, multidrug resistance protein 1 P-glycoprotein; MRP, multidrug resistance–associated protein; NTCP, sodium-taurocholate cotransporting polypeptide; OAT, organic anion transporter; OATP, organic anion transporting polypeptide; SULT, sulfotransferase; UGT, uridine 5'-diphospho-glucuronosyltransferase.*

Supplementary Table 4. Linear mixed effect (LME) model predicted fold change differences from control following 72-hour treatment exposure in sandwich-cultured human hepatocytes (SCHH) shown in comparison to previously published (Vildhede et al., 2020[1]) fold change proteomic data in livers from patients with metabolic dysfunction-associated steatohepatitis (MASH).

Transport Protein	Treatment	LME Model Predicted Mean Fold Change for SCHH Data	Previously Reported Fold Change (MASH vs. Normal Liver) in Vildhede et al.[1]
Uptake Transporters			
OATP1B1	OA:PA	0.72	**0.69**
	LM	0.63	
	LM + 0.01 mM Chol	0.74	
OATP1B3	OA:PA	0.8	**0.39**
	LM	0.81	
	LM + 0.01 mM Chol	0.82	
OATP2B1	OA:PA	0.37	**0.56**
	LM	0.33	
	LM + 0.01 mM Chol	0.53	
NTCP	OA:PA	0.71	**0.65**
	LM	0.67	
	LM + 0.01 mM Chol	0.52	
OAT2	OA:PA	0.90	**0.71**
	LM	0.74	
	LM + 0.01 mM Chol	0.88	
OAT7	OA:PA	1.12	**0.77[b]**
	LM	1.05[a]	
	LM + 0.01 mM Chol	1.11	
Efflux Transporters			
BSEP	OA:PA	0.57	**0.82[b]**
	LM	0.66	
	LM + 0.01 mM Chol	0.66	
MRP2	OA:PA	0.71	**0.71[b]**
	LM	0.59	
	LM + 0.01 mM Chol	0.48	
MRP3	OA:PA	1.23	**1.7**
	LM	1.27	
	LM + 0.01 mM Chol	1.22	
MDR1 P-gp	OA:PA	1.0[a]	**1.3[b]**
	LM	1.05[a]	
	LM + 0.01 mM Chol	0.96[a]	

Data from the current study are reported as LME model predicted mean converted to fold change for treatment vs. control. Fold change data (MASH vs. normal liver) from the Vildhede et al.[1] study are shown for comparison. Unless otherwise indicated, all fold change data demonstrated a statistically significant ($p<0.05$) difference from control. Gray shaded rows indicate SCHH treatments that demonstrate a statistically significant ($p<0.05$) fold change in the opposite direction of that reported in Vildhede et al.[1] LME models (*lmer* package; R version 4.3) were fit for each individual protein using a random effect term on hepatocyte lot to account for inter-lot variability in treatment effects. All treatments contained cytokines (1 ng/mL TNF-α + 1.2 ng/mL IL-6). The Lipid Mix (LM) was

comprised of oleic acid (OA; 167 μM) and palmitic acid (PA; 333 μM) and various lysophospholipids (11.25 μM total).

[a]Data reported in the current study with no statistically significant (p<0.05) difference from control.

[b]Data reported by Vildhede et al.[1] with no statistically significant (p<0.05) difference from control.

BSEP, bile salt export pump; Chol, cholesterol; IL, interleukin; MDR1 P-gp, multidrug resistance protein 1 P-glycoprotein; MRP, multidrug resistance–associated protein; NTCP, sodium-taurocholate cotransporting polypeptide; OAT, organic anion transporter; OATP, organic anion transporting polypeptide; TNF, tumor necrosis factor.

References

1. Vildhede, A., Kimoto, E., Pelis, R.M., Rodrigues, A.D.,and Varma, M.V.S. (2020). Quantitative proteomics and mechanistic modeling of transporter-mediated disposition in nonalcoholic fatty liver disease. *Clin. Pharmacol. Ther.* 107, 1128–1137. doi:10.1002/cpt.1699
